# The Dependence of CNT Aerogel Synthesis on Sulfur-driven Catalyst Nucleation Processes and a Critical Catalyst Particle Mass Concentration

**DOI:** 10.1038/s41598-017-14775-1

**Published:** 2017-11-06

**Authors:** Christian Hoecker, Fiona Smail, Martin Pick, Lee Weller, Adam M. Boies

**Affiliations:** 1 University of Cambridge, Department of Engineering, Cambridge, CB2 1PZ United Kingdom; 2 Q-Flo Limited, BioCity, Pennyfoot Street, Nottingham, NG1 1GF United Kingdom

## Abstract

The floating catalyst chemical vapor deposition (FC-CVD) process permits macro-scale assembly of nanoscale materials, enabling continuous production of carbon nanotube (CNT) aerogels. Despite the intensive research in the field, fundamental uncertainties remain regarding how catalyst particle dynamics within the system influence the CNT aerogel formation, thus limiting effective scale-up. While aerogel formation in FC-CVD reactors requires a catalyst (typically iron, Fe) and a promotor (typically sulfur, S), their synergistic roles are not fully understood. This paper presents a paradigm shift in the understanding of the role of S in the process with new experimental studies identifying that S lowers the nucleation barrier of the catalyst nanoparticles. Furthermore, CNT aerogel formation requires a critical threshold of Fe_x_C_y_ > 160 mg/m^3^, but is surprisingly independent of the initial catalyst diameter or number concentration. The robustness of the critical catalyst mass concentration principle is proved further by producing CNTs using alternative catalyst systems; Fe nanoparticles from a plasma spark generator and cobaltocene and nickelocene precursors. This finding provides evidence that low-cost and high throughput CNT aerogel routes may be achieved by decoupled and enhanced catalyst production and control, opening up new possibilities for large-scale CNT synthesis.

## Introduction

A continuous and scalable one-step production of a macroscopic material composed of carbon nanotubes (CNTs) only became possible by adding sulfur (S) to existing floating catalyst (FC) techniques^1^. The innovative step has led to a process for the production of bulk CNT materials which is now under pilot exploration by at least two companies^2,3^. The dramatic improvements provided by bulk CNT materials in diverse fields such as high strength and toughness composites^4–7^, electrically conductive^8^ and thermally conductive materials^9–12^, and electromagnetic shielding^13^ coupled with the facile synthesis route, means applications research continues to expand worldwide at an increasing rate with new groups joining the field^14–17^. There are many chemical routes to gas phase synthesized CNT aerogels, in the following referred to as CNT aerogels, based around hydrocarbons, aromatic hydrocarbons or alcohols^18^ but all share the common features of a catalyst precursor (typically ferrocene) and a S or elemental group 16 containing compound^19^, essential for aerogel synthesis.

S promotion of CNT growth was first recognized empirically in an arc discharge system, soon after Iijima’s seminal paper giving a detailed characterization of CNTs and CNT growth^20^. Early studies showed that S addition both facilitated CNT synthesis at lower temperatures^21^ and increased the diameter range of the CNTs^22^. Initial hypotheses included S positively influencing the growth kinetics by scavenging blocking groups around the growing edge of CNTs or S species binding to and stabilizing the growing ends of CNTs.

In CVD CNT carpet growth where batch reaction times are typically 15–60 minutes^23^, S is not vital but the effects of S on increasing CNT diameters, numbers of walls and yield are observed in numerous studies^24–29^ and also apply in continuous aerogel spinning systems^30^. The role of S in promoting CNT growth is principally attributed to the formation of an FeS-Fe eutectic either as island-zones on the catalytic nanoparticle surface^25,26^ or the eutectic causing full surface liquefaction of the catalyst nanoparticle^28,31^. The change in the catalytic surface then lowers the activation energy for the nucleation of a CNT due to the decreased free surface energy of the eutectic^26^ and increases carbon diffusion processes, favoring CNT growth^28^. These mechanisms are supported by the observed localization of S on the catalyst surfaces detected by EELS mapping^32,33^ and in modeling using semi-empirical molecular orbital theory^34,35^. In continuous CNT spinning systems where reagents spend a short time at the elevated temperatures needed for CNT growth (typically less than 15 s), S (or an analogue such as Se^19^) becomes an essential reaction promoter to enable continuous aerogel formation.

To date, the roles attributed to S are all associated with its influence on surface-chemistry. In this paper we provide evidence for a hitherto unidentified and equally crucial role of S in catalyst nanoparticle nucleation and, essential for aerogel formation, re-nucleation processes. We previously showed that macroscopic aerogel formation is caused by the re-nucleation of small catalytic nanoparticle droplets from a saturated iron-vapor phase in a pyrolytic-product rich environment, driven by the decreasing-temperature gradient in the downstream portion of a flow-through hot-wall reactor^36^. Herein we use *in-situ* particle measurement techniques to track the influence of S on catalyst nucleation in the key aerogel-forming locations.

Classical nucleation theory predicts that for molecular clusters nucleating from a saturated vapor there is a critical cluster size, and associated Gibbs free energy, below which evaporation of the particle will be favored over particle growth via condensation^37^. While formation of liquid droplets would create a bulk liquid phase of lower chemical potential (and hence lower Gibbs free energy) than the vapor phase, for very small particles the reduction in bulk free energy is exceeded by an increase in surface free energy created at the liquid-vapor interface, driving evaporation. As a result there is a critical cluster size at which particles nucleate whereby the lower chemical potential is greater (in absolute terms) than the increased surface free energy. Data from plasma and flame studies across temperature ranges comparable to our reactor predict that Fe nanoparticles would need to reach a size of 9–12 atoms before the bulk energy reduction balances the surface energy increase^38,39^. In this range, the clusters can then pass over the Gibbs free energy barrier, forming stable particles and continuing to grow via condensation and coagulation (see a fuller explanation of classical nucleation theory in supplementary S8). Our study determines that S lowers the nucleation barrier, as nucleation of particles from the vapor occurs in high concentration at locations of lower supersaturation when S is present in the reaction system. S is known to lower nucleation barriers in other fields such as atmospheric chemistry, where S-species promote nucleation of water droplets^40,41^, and in engine exhaust manifolds where the presence of S from fuel stimulates the formation of solid soot nanoparticulates^42–44^. This is the first time a similar effect has been observed and reported for a FC-CVD system. By lowering the nucleation threshold, S hastens catalyst re-nucleation on the downstream temperature profile shifting the nucleation onset towards the hottest point in the reactor, where the CNT synthesis will occur more rapidly. Thus S is vital in creating a catalyst nanoparticle concentration necessary for a sufficient quantity of long CNTs capable of forming bundles and entangling, leading to spinnable CNT aerogels.

As we have shown previously, bulk aerogel formation occurs as a result of catalyst re-nucleation following catalyst particle evaporation. These findings indicate that aerogel formation should be possible by decoupling the initial catalyst material production and the actual CNT aerogel formation. Rather than the size or number concentration of initially-produced or introduced catalyst nanoparticles defining the production of CNTs in the aerogel, this work tests whether the *mass concentration* of the supplied catalytic material is the key parameter. By successfully synthesizing CNT aerogel using a supply of catalyst nanoparticles from a separate reactor with a mean mobility diameter of *d*_p, mean_ > 100 nm, which is much greater than the diameter of catalyst particles found in the synthesized CNTs (*d*_pCNT, mean_ ≈ 15 nm), we show the independence of the CNT formation on nanoparticle diameter and instead determine the *critical mass concentration* required for aerogel formation. Use of a plasma spark generator (Fe nanoparticle source) as well as cobaltocene and nickelocene precursors in different mass concentrations, proved that critical mass concentrations can be universally applied. While cobaltocene and nickelocene are known precursors for CNT growth^45–47^, we also report, to our knowledge for the first time, the continuous, spinnable CNT aerogel formation from cobalt based catalyst nanoparticles.

Decoupling catalyst production and CNT growth could provide the opportunity for new high-production techniques based on cheap feedstocks for catalyst materials (*e.g*. iron shavings) allowing a much greater choice and control in the catalyst formation steps.

## Results

### Role of S in lowering the catalyst nanoparticle nucleation barrier

**Figure 1:**
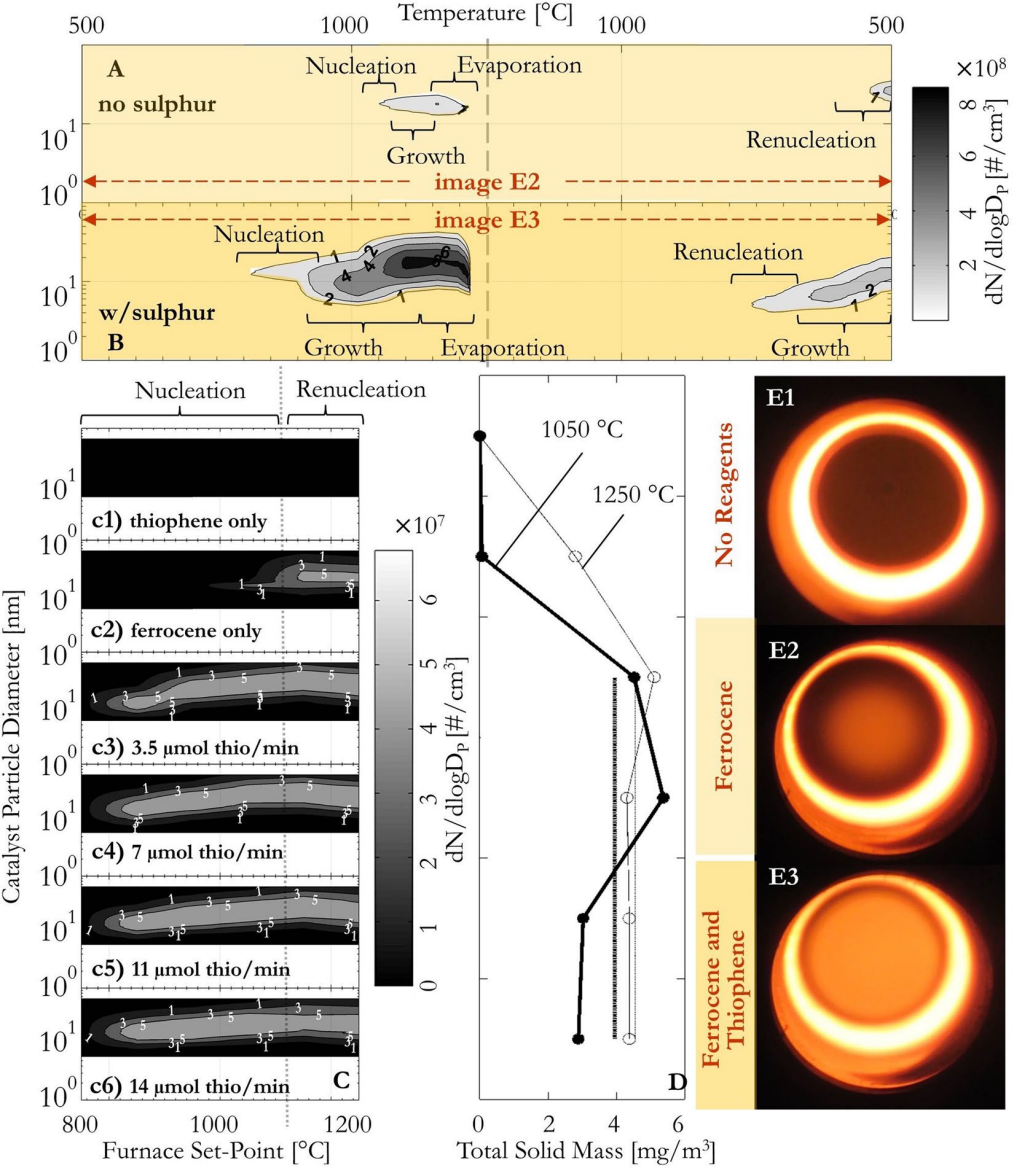
Effect of S on catalyst particle nucleation along the reactor axis. (**A** and **B**) *In-situ* measurements of catalyst particle size distributions along the reactor axis at a furnace set-point of 1250 °C and hydrogen flowrate of 0.5 slpm with only ferrocene (**A**) and ferrocene and thiophene (**B**) entering the furnace. (**c1**–**c6**) Particle size distributions measured at the outlet of the CVD reactor at different furnace temperature set-points and thiophene concentrations with the input of ferrocene kept constant. The distributions shown in (**C**) correspond to set-points 750–1250 °C. Details of all the reactant ratios are given in the Methods section of this paper. (**D**) Total condensed mass of catalyst nanoparticles (Fe_x_C_y_) measured at the outlet at a furnace set-point of 1050 °C and 1250 °C for all reactant concentrations. The straight lines in (**D**) indicate the average total solid mass (Fe_x_C_y_ portion) when thiophene and ferrocene are injected together. (**E1**–**E3**) Photographs of the reactor tube at a furnace set point of 1250 °C showing the absence of catalyst nanoparticles with no reagents (**E1**), a small amount of catalyst nanoparticles from ferrocene injection alone (**E2**) and the increased amount of catalyst nanoparticles on thiophene addition (**E3**).

Ferrocene and thiophene were injected into a FC-CVD reactor at varying concentrations using techniques described in the Methods section of this paper. Particle measurements were conducted along the horizontal reactor axis (Fig. 1A and B) using a reactor set point of 1250 °C and at the reactor *exit* (Figure 1c1–c6) in experiments where the furnace set point was varied between 700–1250 °C. For all data the particle diameter is shown on the vertical axis and number concentrations (d*N*/dlog(*d*_p_)) are indicated by shaded contours with the varying maximum reactor temperatures reported on the horizontal axis (Figure 1c1–c6). The axial measurements in Fig. 1A and B show the nucleation, growth, evaporation and re-nucleation of condensed-phase (liquid or solid) particles within the reactor and contrast the behavior between the presence and absence of S for one precursor ratio. In contrast, Figure 1c1–c6 show the particle size distributions measured at the exit of the reactor for different precursor ratios. The outlet particle size distributions are also converted to a total condensed mass as shown in Fig. 1D. The photographs in Fig. 1E1–E3, looking up the length of the reactor tube, provide qualitative confirmation of the impact of thiophene addition increasing the total particle concentration, reflecting the axial data in Fig. 1A and B.

**Figure 2:**
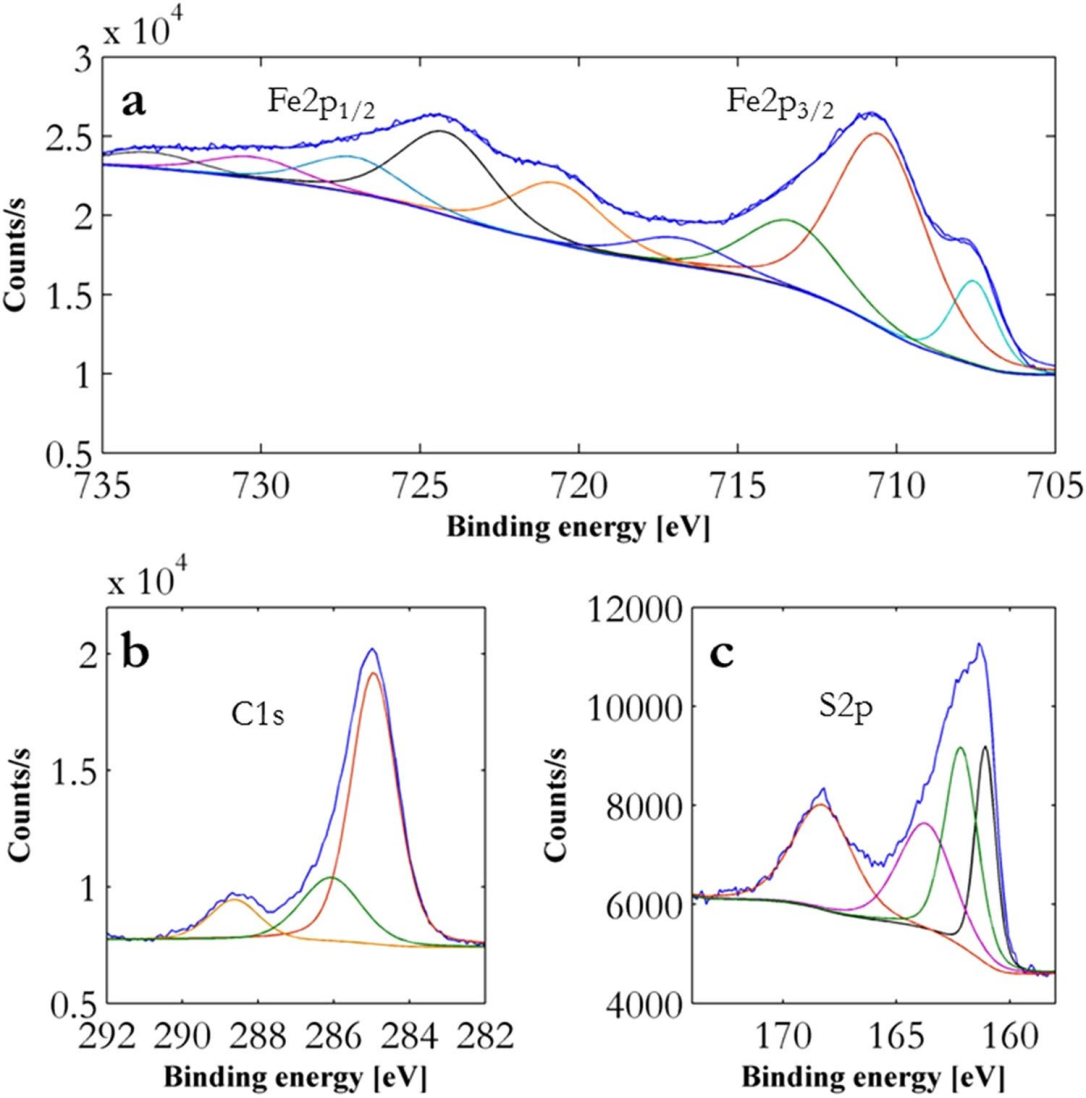
Representative XPS data of catalyst nanoparticles from ferrocene and thiophene at reaction conditions described in the Methods section sampled at the outlet of the reactor. The data shown is for iron (**a**), carbon (**b**) and sulfur (**c**). Raw data in navy blue and all other peaks and background were fitted according to the method described in supplementary S2.

In Fig. 1A axial measurements of the size distribution and concentration of iron-based catalyst nanoparticles formed from ferrocene alone are shown. A parabolic temperature profile exists along the axis, due to furnace end effects, with the hottest point (1250 °C) in the middle of the reactor (typical temperature profile shown elsewhere^36^). Only a small quantity of nanoparticles (total number concentration ~10^6^ #/cm^3^) can be detected upstream of the hottest point at about 1000 °C. No particles can be detected in the hottest zone of the reactor and re-nucleation only occurs very far downstream of the hottest point (~500 °C, total number concentration ~10^8^ #/cm^3^). With the addition of sulfur (Fig. 1B), a higher total concentration of catalyst nanoparticles (total number concentration ~10^9^ #/cm^3^) can be detected further upstream of the hottest point. As in the case of ferrocene alone, no particles are detected in the hottest portion of the reactor. However, both initial nucleation and re-nucleation of the nanoparticles is detected earlier. For example, re-nucleation in Fig. 1B occurs before re-nucleation in Fig. 1A, with a total number concentration at the furnace outlet of ~10^8^ #/cm^3^. These results indicate that a lower supersaturation for catalyst nanoparticle nucleation is necessary in the presence of S for both upstream and downstream locations, and S shifts the catalyst particle re-nucleation towards the hottest location in the reactor. For furnace temperature set-points below 800 °C no nanoparticles can be detected regardless of precursor concentrations and reactant combinations (Figure 1c1–c6) when measuring at the exit of the reactor. Thiophene alone entering the reactor does not form particles at any concentration or furnace set-point temperature used in the experiment (Figure 1c1). However, H_2_S (>10 ppm) is detected in the outlet gas, indicating thiophene decomposition with complete decomposition occurring at temperatures >1000 °C as previously shown using infrared spectroscopy^36^. When only ferrocene enters the reactor, particles form at elevated temperatures (>1050 °C) after its complete decomposition (Figure 1c2). The addition of a trace amount of thiophene (>3.5 µmol/min, concentrations given in the Methods section) leads to a dramatic change in the particle number concentration and total condensed phase mass even at temperatures <1000 °C (Figure 1c3–c6, with an increasing concentration of thiophene from c3–c6). Lowering the nucleation barrier of the catalyst nanoparticles increases the total particle number concentration from ~5 × 10^3^ #/cm^3^ to ~2.5 × 10^7^ #/cm^3^ at 850 °C. Study of the total condensed mass concentration at 1050 °C (Fig. 1D) provides further evidence that the nucleation barrier is lowered in the presence of S, where the total condensed catalyst mass rises from ~0.06 mg/m^3^ to >4 mg/m^3^ on S addition (masses calculated based on the Fe_x_C_y_ portion of the Fe_x_C_y_S_z_ composition indicated by XPS data; method described in supplementary information S2). Using XPS analysis of these particles sampled at the outlet of the reactor, to gain insight into the composition of the catalytic nanoparticles, show that sulfur makes up about 25% of the surface species, by comparing the different peak sizes of the elements (see Fig. 2). Deconvolution of the Fe2p peak suggests contributions from iron oxide and Fe-S species in the nanoparticles, particularly characterized by the peaks observed at 712 and 725 eV^48^.

The nanoparticles formed from ferrocene decomposition alone are a mixture of iron and iron carbide (Fe_x_C_y_), where the detected mass (~0.06 mg/m^3^, first data point in Fig. 1D) is significantly below the total mass of iron available from ferrocene entering the reactor (~10 mg/m^3^, calculated from its vapor pressure curve and assuming full ferrocene-saturation of the entering carrier gas). Once a threshold amount of thiophene (corresponding to ~38 mg/m^3^ atomic sulfur in Figure 1c3) is added, the Fe_x_C_y_ portion of the total condensed mass increases to ~4 mg/m^3^. The Fe_x_C_y_ portion as well as the total condensed mass (including S) does not vary with increasing thiophene concentration. These results indicate that S concentration within particles is likely unaffected by increased thiophene concentrations.

For temperature set-points which are greater than 1050 °C (Figure 1c3–c6) particles initially nucleated after the decomposition of the catalytic precursors will undergo evaporation in the hottest reactor zone. Downstream of the hottest point, the Fe will re-nucleate in the presence of S and C on the downstream temperature profile^36^. At a furnace set-point of 1250 °C, using ferrocene alone as the input, the re-nucleated particles measured at the outlet have a mass concentration (Fe_x_C_y_ portion) of ~2.5 mg/m^3^, a mass concentration of less than 30% of the total mass concentration of iron and carbon supplied. The addition of thiophene at this elevated furnace temperature shows that S is also lowering the nucleation-barrier in the downstream re-nucleation processes as the addition raises the particle concentrations and total outlet condensed masses (Fe_x_C_y_ proportion) up to 65% of the input (Figure 1c3–c6 and B). Calculations show that particle losses due to diffusion and thermophoresis can account for the unrecovered masses (see supplementary S9). Again, further addition of S does not result in additional mass and the Fe_x_C_y_ proportion of the total solid mass fluctuates around an average of ~4.5 mg/m^3^ with comparable particle size distributions having an average particle geometric mean diameter of ~26 nm.

These results also provide insights into previous studies that have suggested the addition of S inhibits the coagulation of catalyst nanoparticles, retarding their growth and therefore promoting the formation of smaller diameter CNTs^33,49–51^. However, our particle size distribution measurements in Fig. 1A,B contradict the assertion that S changes the sticking coefficient of nanoparticles upon collision. Instead results with and without S develop as self-preserving particle size distributions, which are indicative of fully coalescing particles upon collision resulting from particle agglomeration following classical Smoluchowski theory. The particle size distributions reach geometric standard deviations of ~1.46 soon after nucleation (see supplementary information S1). This result is consistent with a sticking coefficient of unity which “is usually the case when submicron aerosol particles collide, provided that they do not carry charges of the same size”^52^. Additionally the coagulation-inhibition hypothesis is contradicted by the almost-immediate coalescence of particles at high temperatures observed in many aerosol studies^53^ and the order of magnitude lower coalescence time for Fe-S when compared to Fe (see supplementary S1).

### Critical mass concentration of Catalyst Material for Aerogel Formation

**Figure 3:**
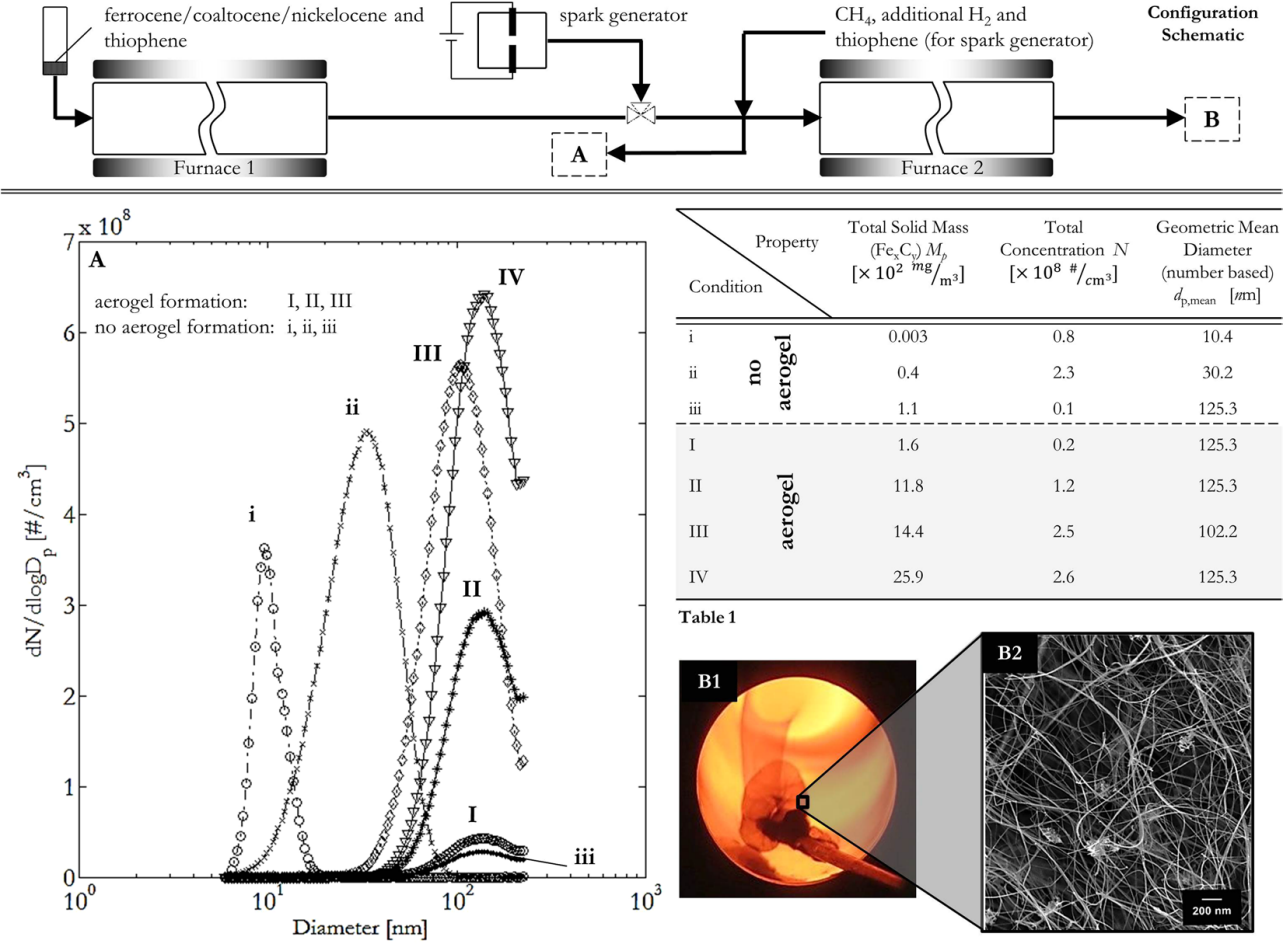
Configuration Schematic (top) Experimental setup for a decoupled CNT aerogel process, measuring the formed nanoparticles after Furnace 1 and collecting CNT material at the outlet of Furnace 2. The temperature set-point of Furnace 1 is varied as described in the Methods, Furnace 2 is set to 1290 °C, a more detailed schematic can be found in the supplementary information (S6). (**A**) Particle size distributions measured at the outlet of Furnace 1 for different temperature set points where (*I – IV*) lead to CNT aerogel formation and (*i – iii*) do not. Further characterization of the nanoparticles entering Furnace 2 is supplied in Table 1. (**B1**,**B2**) Photograph and SEM of a representative CNT aerogel sample taken at the outlet of Furnace 2, feeding in nanoparticles with a mean diameter *d*_p,mean_ > 100 nm (size distributions (*I – IV*)). The aerogel shows CNTs and CNT-bundles of diameter ~8–40 nm with occasional encapsulated-catalyst and carbonaceous impurities.

Having demonstrated that CNT aerogel formation is primarily controlled by S-driven particle re-nucleation on the downward temperature profile, this allows for decoupling of the catalyst supply from the aerogel synthesis. Therefore, CNT aerogel production was carried out by feeding an external supply of catalyst particles into the reactor. The production of catalyst particles was conducted by decomposing thiophene and ferrocene in a separate furnace (Furnace 1 in Fig. 3 – Configuration Schematic) and the catalyst nanoparticles size and mass distributions were characterized at the outlet of Furnace 1. To study the impact of catalyst decoupling on the aerogel formation, different temperatures of Furnace 1 and their effect on measured particle size distributions are shown in Fig. 3B while flow rates and concentrations of reactants entering Furnace 1 were kept constant. Varying the temperature set-point and therefore factors such as decomposition rates of reactants and residence times in the furnace, leads to the generation of different sized catalyst nanoparticles and therefore different total mass concentrations of catalytic material. The catalyst nanoparticles from Furnace 1 then enter Furnace 2 with the addition of CH_4_ and further H_2_ (concentrations given in the Methods section). A set-point of 1290 °C was maintained to facilitate aerogel synthesis. The supply of catalyst particles under certain conditions (*I – IV* in Fig. 3A) led to the formation of a typical aerogel characterized by a wide branched network of CNTs and CNT-bundles of ~8–40 nm diameter with occasional impurities consisting of non-active, carbon-encapsulated catalyst nanoparticles and amorphous carbon material typically found in CVD-synthesized CNT aerogels^54^ (Fig. 3B and Raman supplementary information S10a) whereas the other catalyst particle supplies did not lead to aerogel formation (conditions *i – iii* in Fig. 3A and Table 1 in Fig. 3).

While it is well known that the size of the catalyst strongly influences the diameter of CNTs grown from the catalysts^55^ the SEM analysis of the synthesized CNTs in this set of experiments show that the initial size of the particles (*d*_p, mean_ > 100 nm, distributions *I-IV*) entering Furnace 2 has no impact on the formation and diameter of CNT bundles within the aerogel reactor (diameter 8–40 nm, Fig. 3B2). In fact, BF-STEM studies reveal that catalyst particles of *d*_pCNT, mean_ ≈ 15 nm can be found as being the main promotors for CNT growth in our system. A more detailed study of the catalyst particles and resulting CNT width is presented in the supplementary information S4.

The conditions represented by size distributions in Fig. 3A (i–*iii*) do not lead to CNT aerogel formation despite having very similar total particle number concentration to distributions (*I – II*). In contrast, condition (*III*), which has a total particle number concentration approximately half that of condition (*ii*), does lead to aerogel formation. Counter-intuitively, condition (*i*) with particles that correspond to ideal catalyst sizes for synthesis of CNTs with small numbers of walls (particle diameter 5–15 nm), does not result in aerogel production, while the conditions producing catalyst particles that are much larger (${d}_{{\rm{p}},{\rm{m}}{\rm{e}}{\rm{a}}{\rm{n}}}$ > 100 nm) than ideality for CNT synthesis still result in aerogel formation.

Since the total number concentrations of all particle size distributions are between 0.8–2.6 × 10^8^ #/cm^3^ and larger sized particles result in better aerogel formation, the important metric and difference between the two sets of distributions is the total solid mass. A critical total solid mass concentration of Fe_x_C_y_ of >1.1–1.6 × 10^2^ mg/m^3^ entering Furnace 2 is identified as being the minimum necessary for CNT aerogel formation. Distributions measured downstream of Furnace 1 with a total solid mass of Fe_x_C_y_ less than this critical value (conditions *i, ii* and *iii* in Fig. 3A) did not result in CNT aerogel formation in Furnace 2 regardless of the size and number concentration of the particles. A further experiment was carried out, diluting the particle size distribution shown in (*I*) via splitting of the flow from Furnace 1 and replacing the displaced catalytic-particle containing volume with filtered H_2_. Dilution, resulting in particle size distribution (*III*) corresponding to a total mass concentration of ~1 × 10^3^ mg/m^3^ still produced aerogel as normal. Condition (*IV*, 1.6 × 10^2^ mg/m^3^), which was produced by further diluting condition (*III*) shows the condition with the lowest total solid mass of Fe_x_C_y_ leading to aerogel formation. When the dilution was increased even further so that the catalytic nanoparticle supply dropped in mass concentration (*iii*, 1.1 × 10^2^ mg/m^3^), the aerogel formation ceased, despite the mean diameter of the catalyst nanoparticles in (*iii*) being unchanged from that of (*III*) and (*IV*).

The CNT aerogel formation is therefore not primarily driven by the combination of initial particle number concentration and catalyst nanoparticle diameter. Due to the evaporation and re-nucleation in Furnace 2, the CNT aerogel formation in this process is instead driven by the total solid mass concentration and requires an initial *critical mass concentration* of catalyst material. Therefore, in order to improve the mass production or quality of CNTs with respect to their number, length, diameter or number of tube walls, efforts must be made to influence the downstream catalyst dynamics where the majority of CNTs are formed.

The transient vapor pressure of an iron-carbon-sulfur (Fe_x_C_y_S_x_) catalyst nanoparticle will vary according to the temperature and time profile, as well as catalyst size^56^ and is difficult to determine exactly. However, it is interesting to note that the total equilibrium mass concentration of pure iron in the gas phase at 1290 °C as determined from the empirical vapor pressure relationship (saturation ratio of unity and including the Kelvin effect for 12 nm sized particles)^57^, is ~30 mg/m^3^, correlating closely with our findings (for Fe_x_C_y_), taking into account an estimate of at least 30% particle (mass) losses due to diffusion and thermophoresis (see supplementary S9). If the mass concentration of catalyst material (either in a precursor, vapor or particle) is below the critical value (1.1–1.6 × 10^2^ mg/m^3^), there will be insufficient saturation of the gas and therefore re-nucleation of catalyst nanoparticles at a high concentration at very elevated temperatures in the reactor cannot occur. This will lead to significantly reduced, if any, interaction with the pyrolic species of the carbon source, also identified as being necessary for successful aerogel formation^58^. Therefore, even if a few catalyst nanoparticles nucleate and consequently some CNTs are formed at lower temperatures, insufficient catalyst nanoparticles, and their resulting CNTs form for CNT aerogel formation to occur. Upon examination, our findings are commensurate with all of the published experiments on CNT aerogel formation via a FC-CVD process. Analysis of their data shows the use of Fe_x_C_y_ mass concentrations which are greater than the identified critical mass concentration. An overview of this data is presented in supplementary S5.

### CNT aerogel formation from varying metallic precursors

As nickel (Ni), cobalt (Co) and Fe have similar melting temperatures and vapor pressure curves^56^, the data implies that nanoparticles of these metals will go through similar evaporation and re-nucleation processes along the reactor axis as those derived from ferrocene (compare data of particle size distributions from different precursors as a function of temperature (supplementary S3)). Therefore, to further verify *a*) the critical mass concentration exemplified by the decoupling of catalyst precursor decomposition from the aerogel formation and *b*) the necessity of S in catalytic nanoparticle formation for aerogel synthesis, different catalyst precursors (nickelocene, cobaltocene and iron nanoparticles from a plasma spark generator) were used in combination with methane and tested both with and without thiophene to produce CNTs. Initially, for all of the different metal sources, the total mass of catalyst nanoparticles entering the reactor was chosen to be less than the critical mass concentration required for continuous formation of a CNT aerogel, so that while products formed, equipment blockage was prevented. Particle size distributions were measured for each precursor source and are presented in the supplementary information of this paper (S6). Samples of the product at the outlet of the reaction furnace were collected on a silicon wafer by means of a thermophoretic precipitator.

**Figure 4:**
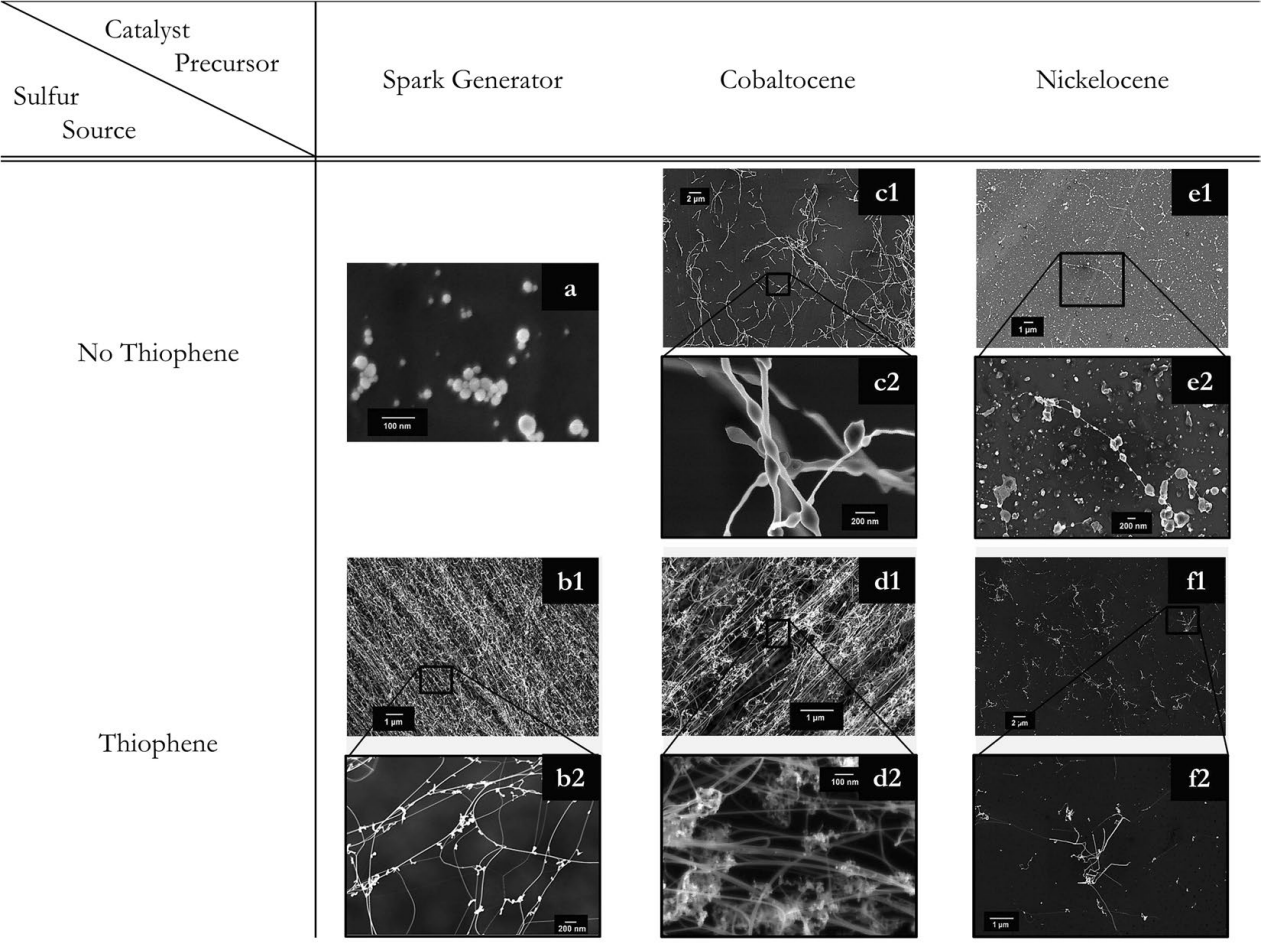
Overview of SEM images of samples resulting from different catalyst precursors – iron nanoparticles from a spark generator (**a**–**b2**), cobalt nanoparticles from cobaltocene (**c1**–**d2**), nickel nanoparticles from nickelocene (**e1**–**f2**) and the addition of thiophene. For all reaction conditions shown, CH_4_ was used as a carbon source. The actual concentrations are given in the Methods section of this paper.

Without S, virtually no (Fig. 4a) or only a few short CNTs (Fig. 4c1,c2,e1 and e2) were collected from the reactor outlet for any of the precursor sources. As in experiments with ferrocene, adding thiophene (3.5 × 10^–5^ mol thiophene/min) to the system had an immediate and pronounced effect on the CNT formation by enhancing CNT growth. The addition resulted in entangled CNT networks with very long CNT bundles, grown from iron nanoparticles from a spark generator and cobalt nanoparticles from a cobaltocene precursor, were found in the outlet region of the reactor (Fig. 4b1,b2,d1 and d2). These had the same SEM characteristics as those forming a spinnable aerogel in Fig. 3 (typical bundle diameters 6–40 nm; length of at least several 100 µm, carbonaceous and encapsulated-catalyst impurities, Raman spectra in supplementary information S10b,c). Adding thiophene to a reaction system with nickel nanoparticles being present had a pronounced effect on the CNT formation as well. There was a change from virtually no CNT formation to the synthesis of small CNT clusters (cluster diameter of ~8 µm) with relatively short, curled CNTs observed (Fig. 4f1 and f2).

**Figure 5:**
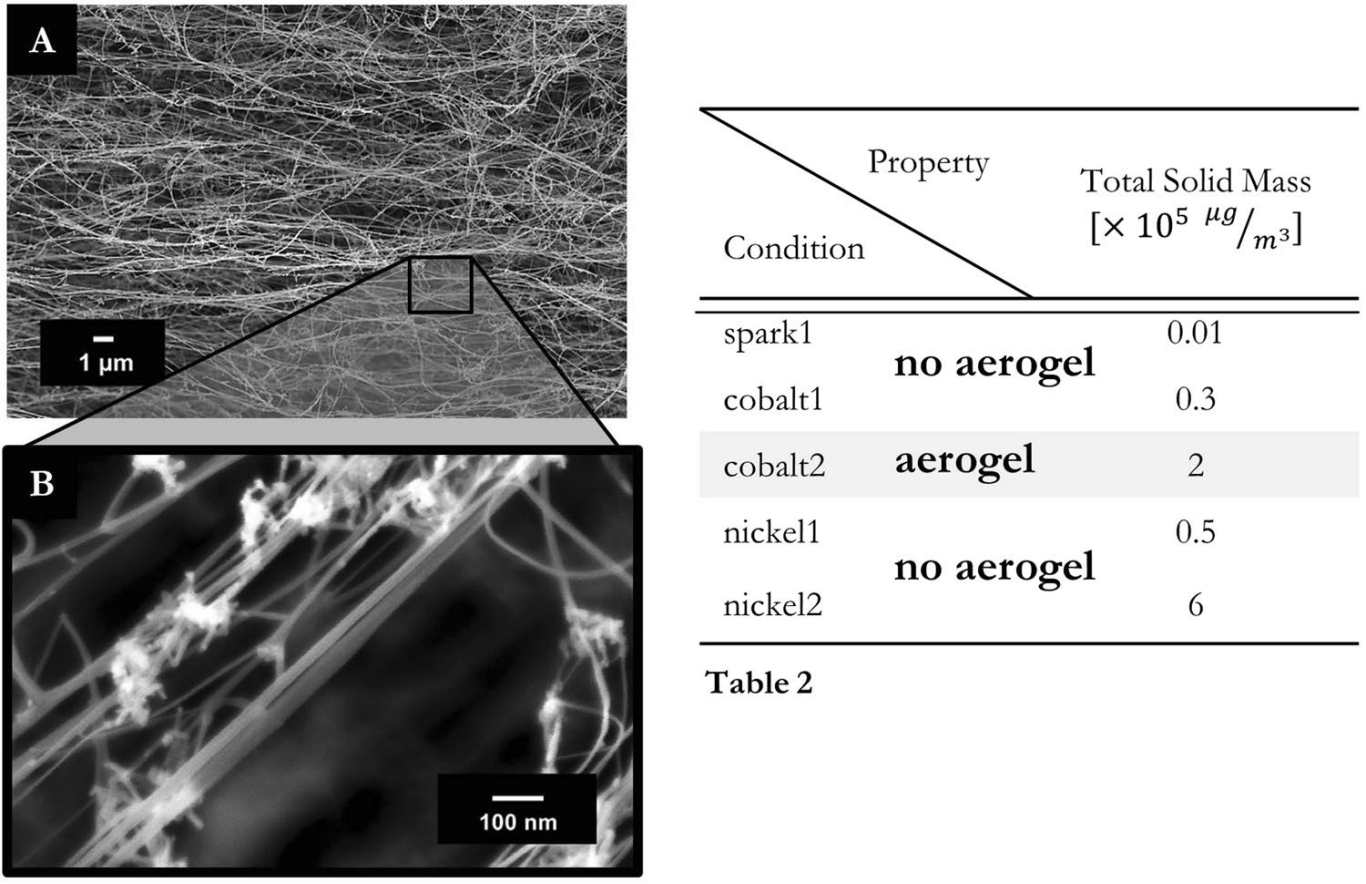
Effect of critical mass concentration on aerogel formation for Fe, Ni and Co sources. (**A** and **B**) SEM analysis of CNT web formed from a cobaltocene precursor showing the same features as that from a ferrocene precursor. Table 2 shows the conditions for spark generated catalyst nanoparticles (spark1), catalyst nanoparticles from cobaltocene (cobalt1 and cobalt2) and catalyst nanoparticles from nickelocene (nickel1 and nickel2) with the addition of thiophene leading to aerogel formation for condition cobalt2 and no aerogel formation for the others due to the total solid mass being below the critical mass concentration for conditions spark1, cobalt1 and nickel1. cobalt2 is at the same time the lowest mass concentration of cobalt capable of aerogel formation and therefore gives a critical mass concentration for cobalt of (>2 × 10^2^ mg/m^3^). For condition nickel2, exceeding (6 × 10^2^ mg/m^3^) an estimated critical mass concentration for nickel, based on the similar vapor pressure curves of cobalt, nickel and iron, no aerogel formation could be observed in our set-up (also see supplementary S6).

To test the critical mass concentration, the total mass concentration of cobalt supplied through cobaltocene was increased to approximately 2 × 10^2^ mg/m^3^, and complete and continuous CNT aerogel formation was achieved (Fig. 5). The total mass from the spark generator could not be increased above the critical mass concentration due to device limitations, preventing formation of a continuous aerogel from this source. However, entangled CNT web was found on the walls of the reactor outlet and, as with decoupled iron-carbon-sulfur particle production in the separate furnaces (Fig. 3 – Configuration Schematic), it is likely that an aerogel would have formed given a mass supply above ~2 × 10^2^ mg/m^3^. In contrast, when further increasing the total mass of nickelocene going into the furnace, no CNT aerogel formation could be achieved and no CNT fiber could be drawn from the reactor, likely due to the difference in the carbon structures grown by the Ni-based catalytic nanoparticles. The significantly smaller CNT clusters growing from the nickel based catalyst nanoparticles (Fig. 4f2 and Raman spectrum supplementary S10d) could not form an entangled CNT network necessary for aerogel formation in our experiments (for further discussion refer to supplementary S3).

## Discussion

This work expands on the previously-reported roles of S enhancing CNT growth and conditioning the catalyst nanoparticles. A major contribution of S is to lower the nucleation barrier of the catalyst nanoparticles themselves. Similar to effects observed in other S containing systems described in the introduction of this paper, S is moving the re-nucleation of catalyst particles to a hotter zone of the reactor where the CNTs can grow to sufficient length and concentration to enable bundling and entanglement and therefore aerogel formation.

The concept of *critical mass concentration* is defined at the entrance of the FC-CVD reaction furnace leading to CNT aerogel formation rather than the process relying on a critical particle number concentration and particle size. Furthermore, it was shown that decoupling the CNT aerogel process is feasible as long as a critical mass concentration of catalyst nanoparticles is supplied to the aerogel synthesis reactor. Combining XPS analysis of the catalyst nanoparticles with particle size distribution and concentration enabled quantification of the critical mass concentration (Fe_x_C_y_ proportion) (1.1–1.6 × 10^2^ mg/m^3^). XPS further identified that at least ~25% of the surface species are S.

The concept of aerogel formation dependence on critical mass concentration is not unique to iron and also holds true for other catalyst precursor sources including cobaltocene, where continuous CNT aerogel formation was achieved from this precursor for the first time.

Combining the flexibility of catalyst decoupling with the new knowledge of S-induced catalyst nucleation increase the possibilities available for use of inexpensive metallic precursors, coupled with innovative reactor design and through these, new possibilities for controlling the purity, morphology and production rates of the final CNT product. Ongoing efforts to produce CNT aerogels at industrial scale may seek to find new methods of decoupled particle production and subsequent CNT growth. The flexibility allowed by this process allows for new synthesis routes from reactors design to optimize the availability of catalyst particle formation in the presence of activated carbon, and thus increase yield, reliability and lower overall production cost.

## Methods

### Investigation of Sulfur-driven Nucleation Processes

To study the effects of S on the catalyst nucleation processes, measurements were carried out by using an alumina (basis 99% Al_2_O_3_) horizontal tube furnace (700 mm × 40 mm ID) at varying temperature set-points of up to 1250 °C and ambient pressure. A schematic of a very similar set-up including a sample probe and a showerhead injector is shown elsewhere^36^. Ferrocene (Acros, purity 98%) and thiophene (Sigma Aldrich, purity ≥99%) as catalyst precursors were diluted in a hydrogen (H_2_ purity grade N5.0, BOC) bulk flow of usually 0.8 slpm, which then entered the reactor tube through a single-point injector.

For the data shown in Fig. 1A and B, ferrocene was supplied via a H_2_ flow (25 sccm ~1 × 10^−6^ mol ferrocene/min) through a sublimation pack set at 85 °C and thiophene was supplied as a vapor in a H_2_ flow (10 sccm, ~1 × 10^−5^ mol thiophene/min). Samples along the centerline were extracted via venturi-suction through a 1.9 mm ID alumina probe with typically a 1:50 dilution with pure and filtered ambient temperature nitrogen at the end of the probe.

For the data shown in Fig. 1c1–6, Ferrocene was supplied via a H_2_ flow (15 sccm, ~4 × 10^−7^ mol ferrocene/min) through a sublimation pack set at 77 °C and thiophene was supplied as a vapor in a H_2_ flow at increasing rates (0, 2.5, 5, 7.5, 10 sccm, corresponding to ~0, 3.5 × 10^−6^, 7 × 10^−6^, 1.1 × 10^−5^, 1.4 × 10^−5^ mol thiophene/min respectively) via a liquid bubbler set at ~0 °C. The injector tip was placed ~10 mm from the inlet of the furnace tube. Particle measurements and classification were conducted from the outlet gas of the reactor. Particles were also investigated by means of XPS and a methods description is included in the supplementary information of this paper (supplementary S2). The samples were collected on a silicon wafer by using a thermophoretic precipitator, a device developed in-house that uses a temperature gradient and resulting thermophoretic forces to efficiently continuously collect particles from an aerosol.

### Investigation of the effect of critical catalyst particle mass concentration on CNT aerogel synthesis

Catalyst particle production was decoupled from CNT aerogel synthesis by using two horizontal furnaces, with catalyst particles generated in the first furnace (Furnace 1) and CNT aerogel formation in the second (Furnace 2). The critical particle mass concentration was identified by characterization of the particles, controlling their concentration entering Furnace 2 and studying their effect on aerogel formation. A schematic of the set-up is shown in Figs 3 and S7 in the supplementary information. In Furnace 1, ferrocene and thiophene were diluted in a H_2_ bulk flow of usually 0.8 slpm (concentration ratios as above) and entered an alumina reactor tube (20 mm ID and 700 mm length) through a single point injector. The temperature set point of Furnace 1 was increased from 850–1150 °C in 100 °C intervals to generate different total particle concentrations and size distributions (distributions in Fig. 3 *i-ii*, *I-II* generated under different Furnace 1 temperature conditions, Fig. 3 *III-IV* and *iii* are dilutions of condition *I*). After cooling the particle stream to room temperature to condense out any remaining ferrocene the classified particles then entered Furnace 2, with a set-point temperature of 1290 °C where methane (CH_4_, N2.5, BOC, 130 sccm) was added along with some additional H_2_ (700–1000 sccm). The removal of varying volumes of catalyst-carrying H_2_ and replacement with filtered H_2_ between Furnace 1 and Furnace 2 allowed dilution of the aerosol coming from Furnace 1. Any CNT aerogel generated was collected on a spinner at the outlet of Furnace 2 through a gas exchange valve as described elsewhere^36^.

### Investigation of critical particle mass concentration and S nucleation effects with different metal sources

A similar equipment configuration as presented for the sulfur work (compare Fig. 3) was used but the metal precursors for the catalyst nanoparticles were varied. Iron nanoparticles generated with an in-house developed spark generator (Fe electrodes, purity >98%, Goodfellow, UK; representative particle size distributions can be found in the supplementary information of this paper, S6) were supplied via a flow of H_2_, cobaltocene was supplied via a H_2_ flow (20–70 sccm, ~3.5–20 × 10^−7^ mol/min) through a sublimation pack set at ~80–90 °C and nickelocene (in similar quantities) was supplied through a similar pack via an argon flow, to prevent its premature decomposition in the supply lines. The supplied catalytic precursor flow of choice was diluted into a bulk flow of H_2_. Thiophene was supplied as a vapor in a H_2_ flow (25 sccm, 3.4 × 10^−5^ mol/min) via a liquid bubbler. The temperatures of the cobaltocene and nickelocene packs were varied to increase their vapor pressures and therefore the total mass delivered to the system.

All of the gas flow rates described throughout were controlled by mass flow controllers (Alicat).

### Analysis and characterization

Particle size and concentration measurements were carried out using a TSI-Scanning Mobility Particle Sizer 3080 (SMPS) system including a TSI-Ultrafine Condensation Particle Counter 3776 (UCPC) and TSI-Differential Mobility Analyzers 3081 and 3085 (DMA). All of the data presented is corrected for dilution, diffusion and thermophoretic losses in the sampling system. SEM (Leo Gemini 1530vp FEG-SEM) was used to study bulk CNT aerogel samples and particles or CNT web collected on silicon wafers. XPS of catalyst particles was carried out on a Thermo Scientific Escalab 250Xi UPS/XPS photoelectronic spectrometer. Scans were recorded with a monochromatic Al Kα anode X-ray source with a power of 210 W, 650 μm spot size and using the adventitious carbon 1 s peak at 284.8 eV as a reference marker to detect sample charging, which was neutralised with an electron flood gun.

Raman analysis (spectra in supplementary information S10) was conducted using a Horiba XploRA PLUS confocal microscope system, using a 638 nm laser, 20x objective, 1200 grating, 1% laser power and 3 accumulations of 30 s. Spectra are presented normalised, with baseline correction applied.

### Availability of datasets

The datasets analyzed in this study are predominately included in this published article and the Supplementary Information. Additional data is available at the following DOI (10.17863/CAM.13668) or from the author on reasonable request.

## Electronic supplementary material

Supplementary Information
