# Supplementary material for: The Dependence of CNT Aerogel Synthesis on Sulfur-driven Catalyst Nucleation Processes and a Critical Catalyst Particle Mass Concentration
Source: Sci Rep. 2017 Nov 6;7:14519. doi: 10.1038/s41598-017-14775-1 (PMC5673953; doi:10.1038/s41598-017-14775-1)

Supplementary Information

Title The Dependence of CNT Aerogel Synthesis on Sulfur-driven Catalyst Nucleation Processes and a Critical Catalyst Particle Mass Concentration

Author(s), and Corresponding Author(s): Christian Hoecker, Fiona Smail, Martin Pick, Lee Weller, Adam M. Boies*

Contents

S1 – EFFECT OF SULFUR ON PARTICLE SIZE DISTRIBUTION 2

S2 – XPS CHARACTERIZATION AND ANALYSIS DETAILS 6

S3 – PARTICLE SIZE DISTRIBUTIONS AT VARIABLE TEMPERATURES 7

S4 - BF-STEM ANALYSIS OF CATALYST NANOPARTICLE AND CNT 10

S5 – MINIMUM MASS CONCENTRATION IN LITERATURE 11

S6 – PARTICLE SIZE DISTRIBUTIONS FOR VARYING CATALYST PRECURSORS 15

S7 – DETAILED SCHEMATIC OF EXPERIMENTAL SET-UP FOR DETERMINING THE CRITICAL MASS CONCENTRATION OF CATALYTIC MATERIAL 16

S8 – CLASSICAL NUCLEATION THEORY - EXPLANATION OF A CRITICAL RADIUS 17

S9 - ESTIMATION OF PARTICLE LOSSES WITHIN REACTOR 21

S10 – RAMAN ANALYSIS OF CNT AEROGELS FROM DIFFERENT CATALYST SOURCES 22

* Corresponding author. Tel: 01223 332600. E-mail: a.boies@eng.cam.ac.uk

S1 – Effect of Sulfur on Particle Size Distribution

In 2011 previous investigators stated that the presence of sulfur may make the iron-based catalytic nanoparticles resistant to coalescence due to the formation of stable Fe-S bonds on the surface [1], citing a reference which contained process modelling to support the theory. Unfortunately, possibly due to an erroneous citation, there is no investigation of the effects of sulfur on catalyst particle size in the cited work[2]. The only earlier work where a similar effect is briefly hypothesised is a paper by Fan et al published in 2000, who noted that the diameter of vapour-grown carbon fibres could be decreased with the addition of "an appropriate amount of sulfur"[3]. The effect hypothesised in 2011 was repeated in a later paper[4] and recent review article[5] with reference to a paper by Lee et. al[6] who made similar claims regarding sulfur-based growth-prevention based on post-experiment analysis of catalyst nanoparticles in their CNT material. In this study we examine the hypothesis that sulfur may reduce the sticking coefficient of colliding particles at temperatures consistent with the CNT process by studying the relative characteristic times of coalescence in Fe and FeS systems and analysing the geometric mean diameter of particle size distributions collected for Fe and Fe-S catalyst nanoparticle systems during the course of our experimental work.

S1a Characteristic Time of Coalescence

The primary growth mechanism of aerosol nanoparticles in a freshly nucleated monodispersed system (*i.e.* one which contains particles of uniform size, such as those nucleating from the decomposition products of precursors) is driven by Brownian motion. The likelihood of two particles sticking together on collision is assumed to be unity as their low

[1] Sundaram, R. M.; Koziol, K. K. K.; Windle, A. H. Continuous Direct Spinning of Fibers of Single-Walled Carbon Nanotubes with Metallic Chirality. *Adv. Mater.* **2011**, *23*, 5064–5068.
[2] Moisala, A.; Nasibulin, A. G.; Kauppinen, E. I. The Role of Metal Nanoparticles in the Catalytic Production of Single-Walled Carbon Nanotubes—a Review. *J. Phys. Condens. Matter* **2003**, *15*, S3011–S3035.
[3] Fan, Y. Y.; Cheng, H. M.; Wei, Y. L.; Su, G.; Shen, Z. H. Tailoring the Diameters of Vapor-Grown Carbon Nanofibers. *Carbon* **2000**, *38*, 921–927.
[4] Paukner, C.; Koziol, K. K. K. Ultra-Pure Single Wall Carbon Nanotube Fibres Continuously Spun without Promoter. *Sci. Rep.* **2014**, 4, 3903.
[5] Janas, D.; Koziol, K. Carbon Nanotube Fibers and Films: Synthesis, Applications and Perspectives of the Direct-Spinning Method. *Nanoscale, 47,* **2016,** 8, *47*, 19475-19490
[6] Lee, K.-H.; Lee, S.-H.; Park, J.; Kim, H.-R.; Lee, J. Synthesis of High-Quality Carbon Nanotube Fibers by Controlling the Effects of Sulfur on the Catalyst Agglomeration during the Direct Spinning Process. *RSC Adv.* **2015**, *5*, 41894–41900.

kinetic energy makes bounce-off unlikely[7]. At high temperatures coalescence is almost instantaneous, driven by the resulting decrease in surface area (-21% for coalescence of two identical diameter spheres) and hence decrease in interfacial energy.[8] The chemistry of the particles, whether they are pure Fe or an Fe-S eutectic does not influence this and their characteristic time of coalescence, τ, in Equation 1 can be calculated based on the temperature dependent viscosity η, the diameter of the particles in question d_p and surface tension σ.

$$\tau = \frac{\eta d_p}{\sigma} \qquad \text{Equation 1}$$

Using the data in Table 1 and assuming particles of the same diameter, the ratio of viscosity to surface tension can be calculated for Fe nanoparticles (3×10^{-6} s/mm) and FeS nanoparticles (4×10^{-7} s/mm). The lower value of the ratio for FeS nanoparticles indicates that these would coalesce with one another much faster than Fe nanoparticles, *i.e.* S does not inhibit "stickiness".

Table 1 : Temperature dependent viscosity and surface tension values for Fe and FeS

	**Fe**	**FeS**	**Notes**
**Viscosity [Pa.s], Temperature [°C]**	0.006, 1527[9]	0.0005, 1527[10]	
**Surface tension [$mN\cdot m^{-1}$], Temperature [°C]**	1817-1935*, 1530[11]	1280, 1525[12]	*Used value of 1876 in calculation

[7] Seinfeld, J.; Pandis, S. Brownian Coagulation. In *Atmospheric Chemistry and Physics From Air Pollution to Climate Change*; Wiley-Interscience: New Jersey, **2006**; pp. 596–598.
[8] Lehtinen, K. E. J.; Zachariah, M. R. Control of Primary Particle Size and the Onset of Aggregate Formation: The Effect of Energy Release in Nanoparticle Collision and Coalescence Processes. *Model. Numer. Simul. Mater. Behav. Evol.* **2002**, *731*, 163–168.
[9] Assael, M. J.; Kakosimos, K.; Banish, R. M.; Brillo, J.; Egry, I.; Brooks, R.; Quested, P. N.; Mills, K. C.; Nagashima, A.; Sato, Y.; *et al.* Reference Data for the Density and Viscosity of Liquid Aluminum and Liquid Iron. *J. Phys. Chem. Ref. Data* **2006**, *35*, 285–300.
[10] Kono, Y.; Kenney-Benson, C.; Shibazaki, Y.; Park, C.; Shen, G.; Wang, Y. High-Pressure Viscosity of Liquid Fe and FeS Revisited by Falling Sphere Viscometry Using Ultrafast X-Ray Imaging. *Phys. Earth Planet. Inter.* **2015**, *241*, 57–64.
[11] Keene, B. J. Review of Data for the Surface Tension of Pure Metals. *Int. Mater. Rev.* **1993**, *38*, 157–192.
[12] Mcnallan, M. J.; Debroy, T. Effect of Temperature and in Fe-Ni-Cr Alloys Containing Sulfur. *Metall. Trans. B* **1991**, *22*, 557-560.

S1b Analysis of the geometric mean diameter of Fe and Fe-S catalyst nanoparticle systems

In our data, we find that sulfur has no retarding effect on the growth of catalytic nanoparticles via coagulation and particle growth follows the principal above. Looking at the data presented in Figure 1 in the main body of this paper, the particle size distributions resulting from ferrocene only and a system including ferrocene and thiophene, show the same general behaviour. As predicted by classical nucleation theory, the geometric mean diameter grows at an identical rate in both cases and in the manner expected for coagulating particles (compare Figure S1i).

In any monodispersed system, after a certain time, particle size distributions of aerosols undergoing coagulation by Brownian motion attain a shape that is invariant with time. If for example the size distribution is scaled by the average particle volume, the distributions map with one other and are therefore described as self-preserving. Self-preserving distributions of coalescing metal nanoparticles obtain a geometric standard deviation (GSD) in their diameter of *i)* σ_g = ~1.46 in the free molecular regime ($d_p << \lambda$, mean free path of the gas) and *ii*) σ_g = ~1.44 ($d_p >> \lambda$) in the continuum regime[13,14]. Particles that grow via condensational growth, absent a particle-particle collisional process, do not attain a self-preserving distribution and retain a σ_g that is near the original distribution. In our data, for a sulfur containing system, we find that σ_g~1.45, being in line with particles in the transition regime ($d_p \sim \lambda$), after a certain distance (and therefore time of travel) from the hottest point of the reactor and after re-nucleation (compare Figure S1ii.). We therefore do not observe any 'sticking' preventing phenomena in sulfur containing systems (which would be indicated by $\sigma_g$values significantly below 1.46) as stated by other researchers.

[13] Landgrebe, J. D.; Pratsinis, S. E. Gas-Phase Manufacture of Particulates: Interplay of Chemical Reaction and Aerosol Coagulation in the Free-Molecular Regime. *Ind. Eng. Chem. Res.* **1989**, *28*, 1474–1481.
[14] Granqvist, C. G.; Buhrman, R. A. Ultrafine Metal Particles. *J. Appl. Phys.* **1976**, *47*, 2200–2219.

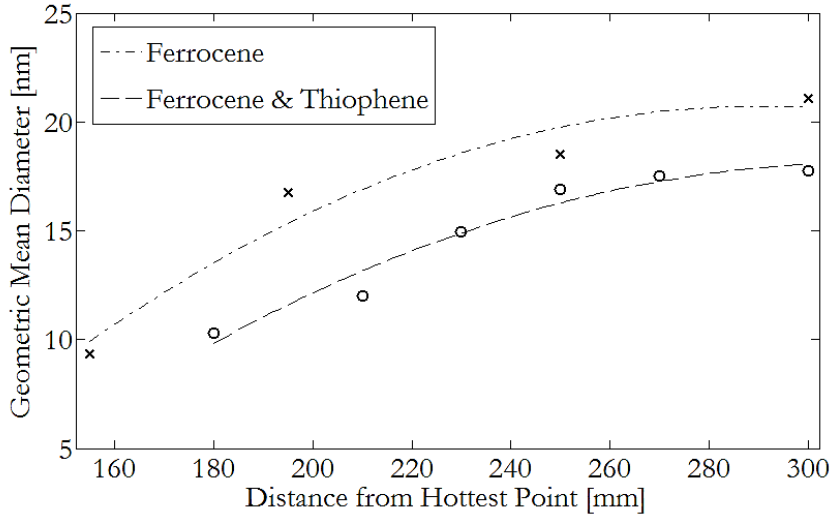

Figure S1i. Geometric mean diameter of ferrocene only and ferrocene & thiophene containing systems as a function of distance from the hottest point in the reactor. The rates of increase in diameter are approximately the same for each system.

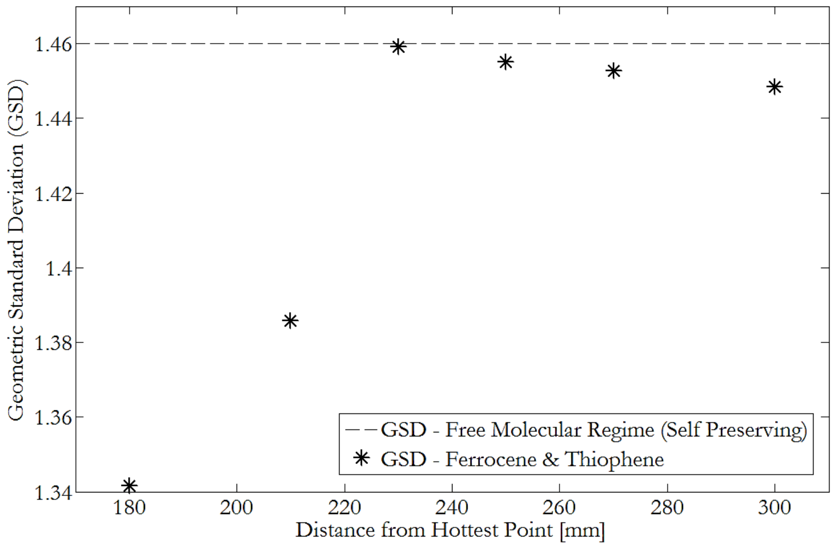

Figure S1ii. Geometric standard deviation (GSD) for a system containing ferrocene and thiophene. 220 mm downstream from the hottest point of the reactor, the particle size distribution reaches a GSD of 1.46. We find no evidence of a 'non-sticking phenomena' occurring, which we would expect to observe as a lower GSD.

S2 – XPS Characterization and Analysis Details[15]

The data presented in Figure 2 of the main paper shows a representative set of data of the iron (Fe), carbon (C) and sulfur (S) peaks from X-ray photoemission spectroscopy (XPS) analysis of catalytic nanoparticles collected on a silicon (Si) wafer at the furnace exit using a thermophoretic precipitator, an in-house device that uses a temperature gradient and resulting thermophoretic forces to collect >90% all of the particles from the aerosol irrespective of diameter. The samples were analyzed using a Thermo Scientific Escalab 250Xi UPS/XPS photoelectronic spectrometer. Scans were recorded with a monochromatic Al Kα anode X-ray source with a power of 210 W, 650 μm spot size and using the adventitious carbon 1s peak at 284.8 eV as a reference marker to detect sample charging, which was neutralised with an electron flood gun. XPS spectra were collected from an area of 300 μm^2 on the Si substrate, with an interaction depth estimated to ~10 nm. XPS data was interpreted using the Thermo Scientific Avantage software. For each sample a survey scan was first carried out, followed by high resolution scans on the spectral regions of interest. A total amount of at least ~25% of sulfur on the surface of the nanoparticle can be identified by comparing the different peak sizes of the elements. No CH_4 was present in these experiments so that all of the carbon detected was coming from either ferrocene or thiophene and their decomposition products. Therefore, the results might only be partially transferable to a complete CNT aerogel reaction system (*e.g.* actual CNT growth composition). The total catalyst nanoparticle surface composition ($Fe_xC_yS_z$) is calculated via XPS. We choose to ignore the S content in our total mass concentration calculations because our results show that increasing S concentration, above a minimal amount, does not influence nucleation phenomena and particle composition. Therefore, the data is presented in terms of the Fe_xC_y proportion of the particle composition, which is the important measure for the critical mass concentration necessary for CNT aerogel formation reported in the latter section of the main paper.

[15] Acknowledgements to *Jean de la Verpillière*, University of Cambridge for helping with the description.

S3 – Particle Size Distributions at Variable Temperatures

To investigate the effect of the critical mass concentration on the behaviour of different catalyst materials, experiments similar to the ones described in the Methods section of the main paper body were conducted by sampling nickel based catalyst nanoparticles from a nickelocene precursor and iron based nanoparticles from a spark generator, with and without the addition of sulfur, from the hottest point of the furnace tube across a temperature range of 800–1350 °C. In Figure S3i and Figure S3ii it can be seen that, very similar to as described elsewhere[16], no nanoparticles could be observed at very elevated temperatures (>1200 °C) due to the evaporation of the nanoparticles.

Nickelocene has a very similar vapour pressure curve to ferrocene[17] and was supplied as a vapour from a sublimator, with argon used as a carrier gas through the sublimator. This is because, due to the greater instability of nickelocene[18], it reacts with H_2 at temperatures greater than 50 °C[19]. However, to replicate the conditions usually used for CNT growth, the Ar-nickelocene stream had to be mixed with a bulk H_2 flow through the injector system, resulting in very early decomposition compared to the ferrocene analogue. Therefore, for the nickel based catalyst nanoparticles, high concentrations of nanoparticles can be observed without any addition of sulfur (mean diameter $d_{\text{p, nickel, mean}} = \sim 6$ nm, total number concentration $\sim 6\times10^7$ #/cm^3, total solid mass < 0.1 mg/m^3) as in this case nucleation will occur very rapidly due to the inherent instability of the nickelocene molecule at the low temperatures leading to a high supersaturation of the gas[19] (Figure S3i). This contrasts with results from ferrocene presented in Figure 1 of the main paper. Addition of thiophene (~5 µmol/min) to the nickelocene system (Figure S3i.b.) resulted in little change in the total particle concentration in the hottest zone of the reactor, as the

[16] Hoecker, C.; Smail, F.; Bajada, M.; Pick, M.; Boies, A. Catalyst Nanoparticle Growth Dynamics and Their Influence on Product Morphology in a CVD Process for Continuous Carbon Nanotube Synthesis. *Carbon.* **2016**, *96*, 116–124.

[17] Vieyra-Eusebio, M. T.; Rojas, A. Vapor Pressures and Sublimation Enthalpies of Nickelocene and Cobaltocene Measured by Thermogravimetry. *J. Chem. Eng. Data* **2011**, *56*, 5008–5018.

[18] Dyagileva, L. M.; Mar'in, V. P.; Tsyganova, E. I. Reactivity of the First Transition Row Metallocenes in Thermal Decomposition Reaction. *J. Organomet. Chem.* **1979**, *175*, 63–72.

[19] Dormans, G. J. M. OMCVD of Transition Metals and Their Silicides Using Metallocenes and (Di) Silane or Silicon Tetra-Bromide. *J. Cryst. Growth* **1991**, *108*, 806–816 and Brissonneau, L.; Sahnoun, R.; Mijoule, C.; Vahlas, C. Investigation of Nickelocene Decomposition during Chemical Vapor Deposition of Nickel. *J. Electrochem. Soc.* **2000**, *147*, 1443.

incoming gas was already carrying nucleated nanoparticles due to the instability of nickelocene in hydrogen and slightly elevated temperatures.

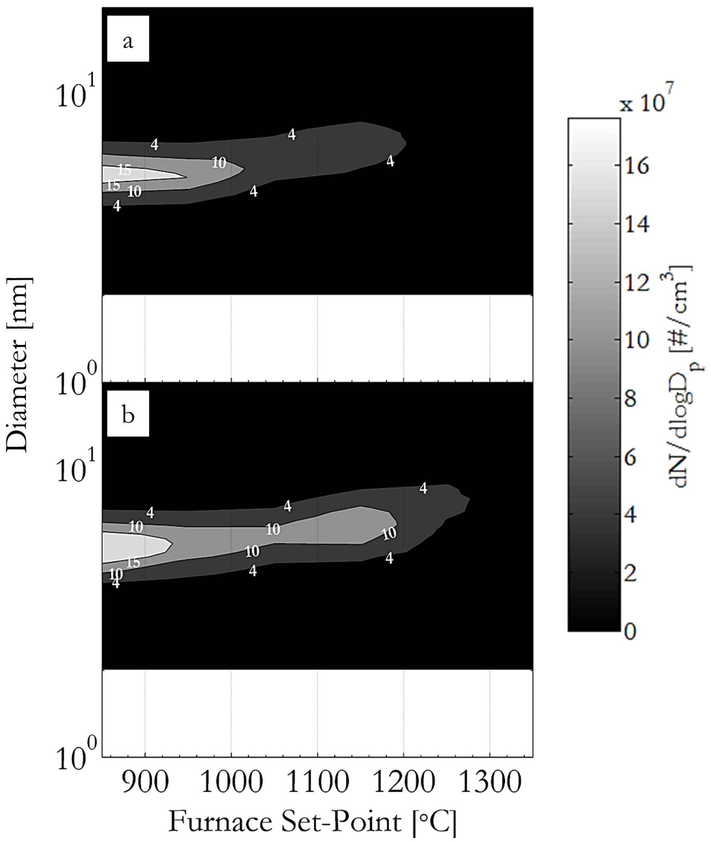

Figure S3i. Particle size distributions of nickel based catalyst nanoparticles taken from the hottest location in the reactor at variable temperatures (up to 1350 °C) without (a) and with (b) the addition of thiophene (~5 µmol/min).

For iron based particles generated from a spark generator a very similar behaviour to those from ferrocene is observed (including their behaviour when S is added, Figure S3ii.). Solid particles produced from iron vapour and thiophene with a mean diameter of $d_{\mathrm{p\ spark,mean}}$ = ~15 nm (total concentration ~4×10^6 #/cm^3, total solid mass ~ 1 mg/m^3, which is below the critical mass of ~110 – 160 mg/m^3 required for aerogel formation) from the spark generator enter the furnace and evaporate at the elevated temperatures.

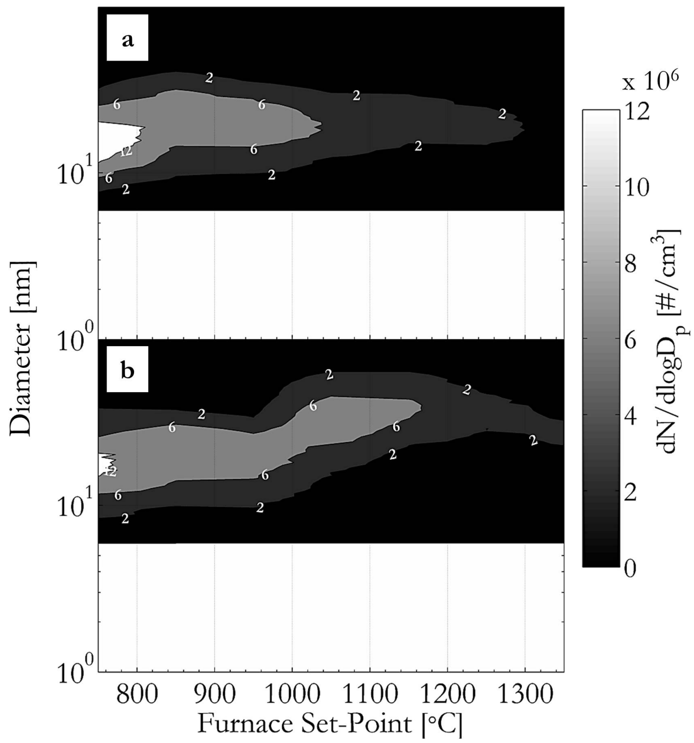

Figure S3ii. Particle size distributions of iron based catalyst nanoparticles taken from the hottest location in the reactor at variable temperatures (up to 1350 °C) without (a) and with (b) the addition of thiophene (10 μmol/min).

S4 - BF-STEM Analysis of Catalyst Nanoparticle and CNT

The CNTs and CNT bundles shown in the body of the main paper and in Figure S4.a are much smaller than the diameter of catalyst nanoparticles ($d_{p,mean}$ > 100 nm) introduced into Furnace 2 after their separate synthesis in Furnace 1. As shown in Figure S4.b and c, the catalyst nanoparticles leading to CNT formation are on average about 20 nm in diameter. The samples were prepared by sonication of the formed CNT aerogel in ethanol for >10 minutes, followed by dropping the solution onto lacey carbon TEM grids and allowing the solvent to evaporate. Analysis was then carried out on a Hitachi S-5500 In-Lens FE SEM (2009) and 15.0 kV was used for the BF-STEM images

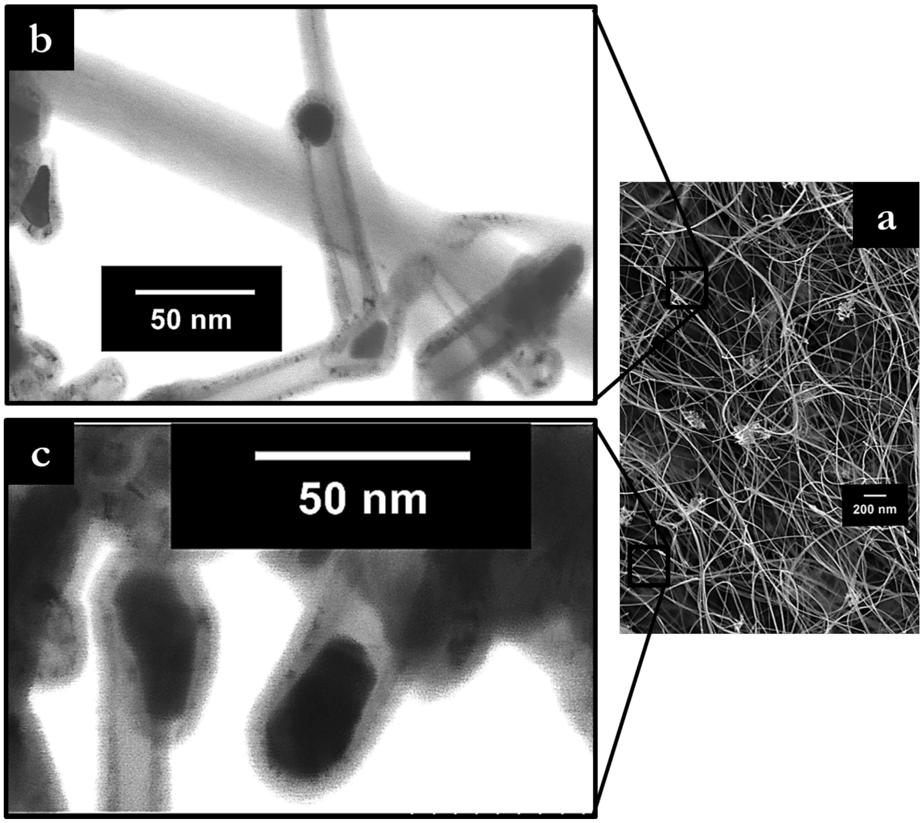

Figure S4. BF-STEM analysis of CNT web formed at the outlet of Furnace 2 in the decoupled process. The catalyst nanoparticles leading to CNT formation are usually about 20 nm wide as shown in b) and c), as representative images, and therefore much smaller than the catalyst nanoparticles introduced from Furnace 1.

S5 – Minimum Mass Concentration in Literature

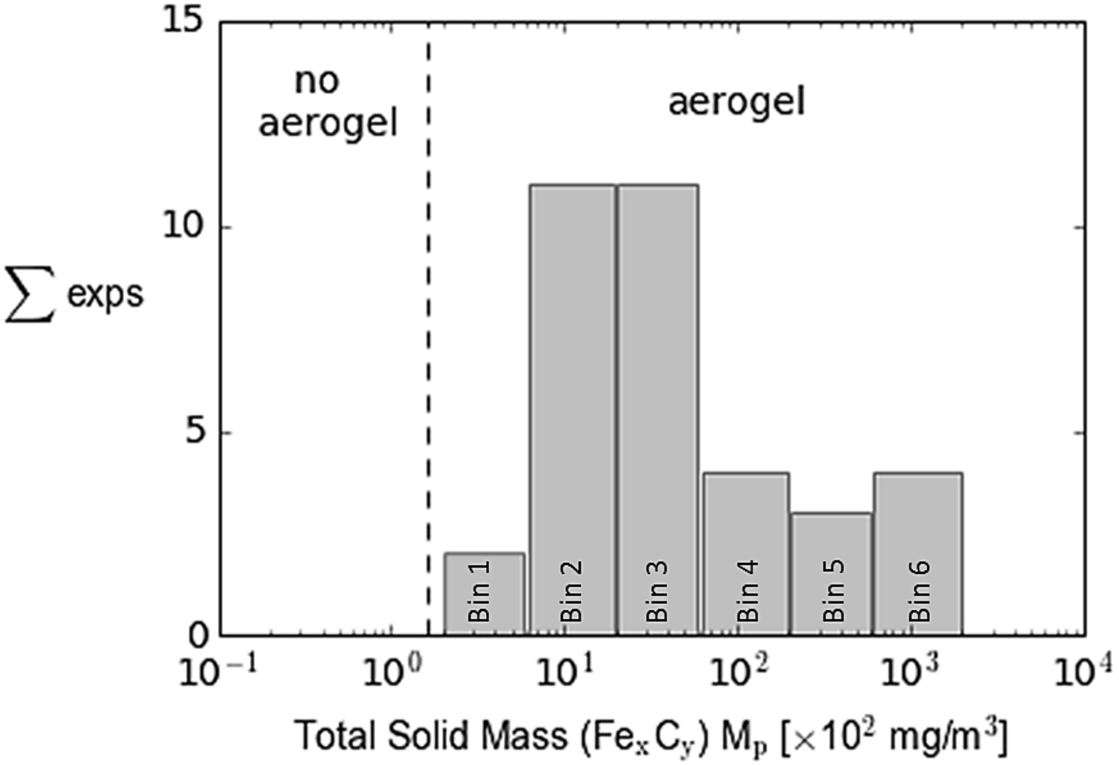

Figure S5. Bin 1 – 6 show total solid mass input for elemental Iron and Carbon calculated from the catalyst and promoter sources in published successful FCCVD experiments (see reference list below). The black (dotted) line corresponds to the identified minimum mass concentration (1.6×10^2 mg/m^3) required for successful aerogel formation. This value was determined experimentally from Figure 3 in the main text. The total solid mass (mg/m^3) for each experiment is the ratio of elemental mass per unit time (mg/s) to total carrier gas flow rate (m^3/s). The number of experiments are binned within certain ranges of masses. Most experiments (Bin 2 and 3) use an order of magnitude larger Fe_xC_y content than the required minimum mass concentration.

List of References

Bin 1

1. Alemán, B.; Reguero, V.; Mas, B.; Vilatela, J. J. Strong Carbon Nanotube Fibers by Drawing Inspiration from Polymer Fiber Spinning. *ACS Nano* **2015**, *9*, 7392–7398.
2. Mas, B.; Alemán, B.; Dopico, I.; Martin-Bragado, I.; Naranjo, T.; Pérez, E. M.; Vilatela, J. J. Group 16 Elements Control the Synthesis of Continuous Fibers of Carbon Nanotubes. *Carbon* **2016**, *101*, 458–464.

Bin 2

1. Motta, M.; Kinloch, I.; Moisala, A.; Premnath, V.; Pick, M.; Windle, A. The Parameter Space for the Direct Spinning of Fibres and Films of Carbon Nanotubes. *Phys. E Low-dimensional Syst. Nanostructures* **2007**, *37*, 40–43.
2. Zhong, X.-H.; Li, Y.-L.; Liu, Y.-K.; Qiao, X.-H.; Feng, Y.; Liang, J.; Jin, J.; Zhu, L.; Hou, F.; Li, J.-Y. Continuous Multilayered Carbon Nanotube Yarns. *Adv. Mater.* **2010**, *22*, 692–696.
3. Conroy, D.; Moisala, A.; Cardoso, S.; Windle, A.; Davidson, J. Carbon Nanotube Reactor: Ferrocene Decomposition, Iron Particle Growth, Nanotube Aggregation and Scale-Up. *Chem. Eng. Sci.* **2010**, *65*, 2965–2977
4. Gspann, T.; Smail, F.; Windle, A. Spinning of Carbon Nanotube Fibres Using the Floating Catalyst High Temperature Route: Purity Issues and the Critical Role of Sulfur. *Faraday Discuss.* **2014**, *173*, 2–7. (2 datapoints)
5. Reguero, V.; Alemán, B.; Mas, B.; Vilatela, J. J. Controlling Carbon Nanotube Type in Macroscopic Fibers Synthesized by the Direct Spinning Process. *Chem. Mater.* **2014**, *26*, 3550–3557. (2 datapoints)
6. Hoecker, C.; Smail, F.; Bajada, M.; Pick, M.; Boies, A. Catalyst Nanoparticle Growth Dynamics and Their Influence on Product Morphology in a CVD Process for Continuous Carbon Nanotube Synthesis. *Carbon* **2016**, *96*, 116–124.
7. Alemán, B.; Reguero, V.; Mas, B.; Vilatela, J. J. Strong Carbon Nanotube Fibers by Drawing Inspiration from Polymer Fiber Spinning. *ACS Nano* **2015**, *9*, 7392–7398. (2 datapoints)
8. Lee, K.-H.; Lee, S.-H.; Park, J.; Kim, H.-R.; Lee, J. Synthesis of High-Quality Carbon Nanotube Fibers by Controlling the Effects of Sulfur on the Catalyst Agglomeration during the Direct Spinning Process. *RSC Adv.* **2015**, *5*, 41894–41900.

Bin 3

1. Li, Y.-L.; Kinloch, I. a; Windle, A. H. Direct Spinning of Carbon Nanotube Fibers from Chemical Vapor Deposition Synthesis. *Science* **2004**, *304*, 276–278.
2. Wei, J.; Jiang, B.; Wu, D.; Wei, B. Large-Scale Synthesis of Long Double-Walled Carbon Nanotubes. *J. Phys. Chem. B* **2004**, *108*, 8844–8847.
3. Conroy, D.; Moisala, A.; Cardoso, S.; Windle, A.; Davidson, J. Carbon Nanotube Reactor: Ferrocene Decomposition, Iron Particle Growth, Nanotube Aggregation and Scale-Up. *Chem. Eng. Sci.* **2010**, *65*, 2965–2977

4. Sundaram, R. M.; Koziol, K. K. K.; Windle, A. H. Continuous Direct Spinning of Fibers of Single-Walled Carbon Nanotubes with Metallic Chirality. *Adv. Mater.* **2011**, *23*, 5064–5068.
5. Zhong, X.-H.; Li, Y.-L.; Feng, J.-M.; Kang, Y.-R.; Han, S.-S. Fabrication of a Multifunctional Carbon Nanotube "cotton" Yarn by the Direct Chemical Vapor Deposition Spinning Process. *Nanoscale* **2012**, *4*, 5614–5618.
6. Zhong, X.; Wang, R.; Yangyang, W.; Yali, L. Carbon Nanotube and Graphene Multiple Thread Yarns. *Nanoscale* **2013**, *5*, 1183–1187.
7. Jung, Y.; Song, J.; Huh, W.; Cho, D.; Jeong, Y. Controlling the Crystalline Quality of Carbon Nanotubes with Processing Parameters from Chemical Vapor Deposition Synthesis. *Chem. Eng. J.* **2013**, *228*, 1050–1056. (3 datapoints)
8. Xu, H.; Tong, X.; Zhang, Y.; Li, Q.; Lu, W. Mechanical and Electrical Properties of Laminated Composites Containing Continuous Carbon Nanotube Film Interleaves. *Compos. Sci. Technol.* **2016**, *127*, 113–118.
9. Hou, G.; Su, R.; Wang, A.; Ng, V.; Li, W.; Song, Y.; Zhang, L.; Sundaram, M.; Shanov, V.; Mast, D.; *et al.* The Effect of a Convection Vortex on Sock Formation in the Floating Catalyst Method for Carbon Nanotube Synthesis. *Carbon.* **2016**, *102*, 513–519.

Bin 4

1. Li, Y.-L.; Kinloch, I. A; Windle, A. H. Direct Spinning of Carbon Nanotube Fibers from Chemical Vapor Deposition Synthesis. *Science* **2004**, *304*, 276–278.
2. Li, Y.-L.; Zhang, L.-H.; Zhong, X.-H.; Windle, A. H. Synthesis of High Purity Single-Walled Carbon Nanotubes from Ethanol by Catalytic Gas Flow CVD Reactions. *Nanotechnology* **2007**, *18*, 225604. (2 datapoints)
3. Park, Y.-S.; Huh, M.-Y.; Kang, S.-J.; Lee, S.-H.; An, K.-H. Parametric Study on Synthesis of Carbon Nanotubes by the Vertical Spray Pyrolysis Method. *Carbon Lett.* **2011**, *12*, 102–106.

Bin 5

1. Feng, J.-M.; Wang, R.; Li, Y.-L.; Zhong, X.-H.; Cui, L.; Guo, Q.-J.; Hou, F. One-Step Fabrication of High Quality Double-Walled Carbon Nanotube Thin Films by a Chemical Vapor Deposition Process. *Carbon* **2010**, *48*, 3817–3824.
2. Lee, K.-H.; Lee, S.-H.; Park, J.; Kim, H.-R.; Lee, J. Synthesis of High-Quality Carbon Nanotube Fibers by Controlling the Effects of Sulfur on the Catalyst Agglomeration during the Direct Spinning Process. *RSC Adv.* **2015**, *5*, 41894–41900.
3. Wu, T.; Wang, J. N. Carbon Nanotube Springs with High Tensile Strength and Energy Density. *RSC Adv.* **2016**, *6*, 38187–38191.

Bin 6

1. Wang, J. N.; Luo, X. G.; Wu, T.; Chen, Y. High-Strength Carbon Nanotube Fibre-like Ribbon with High Ductility and High Electrical Conductivity. *Nat. Commun.* **2014**, *5*, 1-8
2. Xu, W.; Chen, Y.; Zhan, H.; Wang, J. N. High-Strength Carbon Nanotube Film from Improving Alignment and Densification. *Nano Lett.* **2016**, *16*, 946–952.

3. Lee, S.-H.; Park, J.; Kim, H.-R.; Lee, T.; Lee, J.; Im, Y.-O.; Lee, C.-H.; Cho, H.; Lee, H.; Jun, C.-H.; *et al.* Synthesis of Carbon Nanotube Fibers Using the Direct Spinning Process Based on Design of Experiment (DOE). *Carbon* 2016, *100*.
4. Lee, K.-H.; Lee, S.-H.; Park, J.; Kim, H.-R.; Lee, J. Synthesis of High-Quality Carbon Nanotube Fibers by Controlling the Effects of Sulfur on the Catalyst Agglomeration during the Direct Spinning Process. *RSC Adv.* 2015, *5*, 41894–41900.

S6 – Particle Size Distributions for varying Catalyst Precursors

Figure S6 shows the corresponding catalyst particle size distributions which resulted in the morphologies of the CNT species shown in Figure 4 of the main paper. The particles were sampled at the furnace exit using an SMPS fitted with either a Long- (used to characterize particles up to ~300 nm) or a Nano- (used to characterize particles up to ~80 nm) DMA as described in the Methods section within the main body of the paper. Entangled CNTs and CNT bundles were observed from iron spark generator and cobaltocene generated nanoparticles with the addition of thiophene. Nickelocene showed only small CNT clusters whether thiophene was added or not. Macroscopic CNT aerogel formation was achieved when increasing the total solid mass of cobalt going into the reaction furnace above a mass concentration of 200 mg/m^3.

Catalyst Precursor / Sulfur Source	Spark Generator	Cobaltocene	Nickelocene
Particle Size Distribution<br>···⊙··· no thiophene<br>···×··· thiophene			

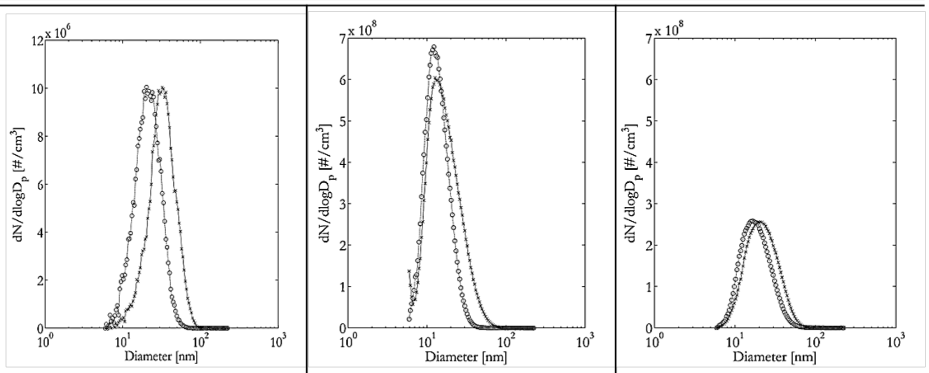

	No thiophene	thiophene		No thiophene	thiophene		No thiophene	thiophene
Total Concentration [$\#/cm^3$]	4.2×10^6	4.1×10^6	Total Concentration [$\#/cm^3$]	2.5×10^8	3.0×10^8	Total Concentration [$\#/cm^3$]	1.2×10^8	1.3×10^8
Total Solid Mass [mg/m^3]	0.3	1.0	Total Solid Mass [mg/m^3]	5	17	Total Solid Mass [mg/m^3]	8	15

Figure S6. Particle size distributions measured at the outlet of the furnace (furnace set-point temperature 1250 °C) with and without thiophene. The total solid mass reported is assuming that the particles are entirely composed of the respective transition metal (iron, cobalt, nickel). The data has therefore not been corrected for the actual particle composition as done in the main body of the paper.

S7 – Detailed Schematic of Experimental Set-Up for determining the Critical Mass Concentration of Catalytic Material

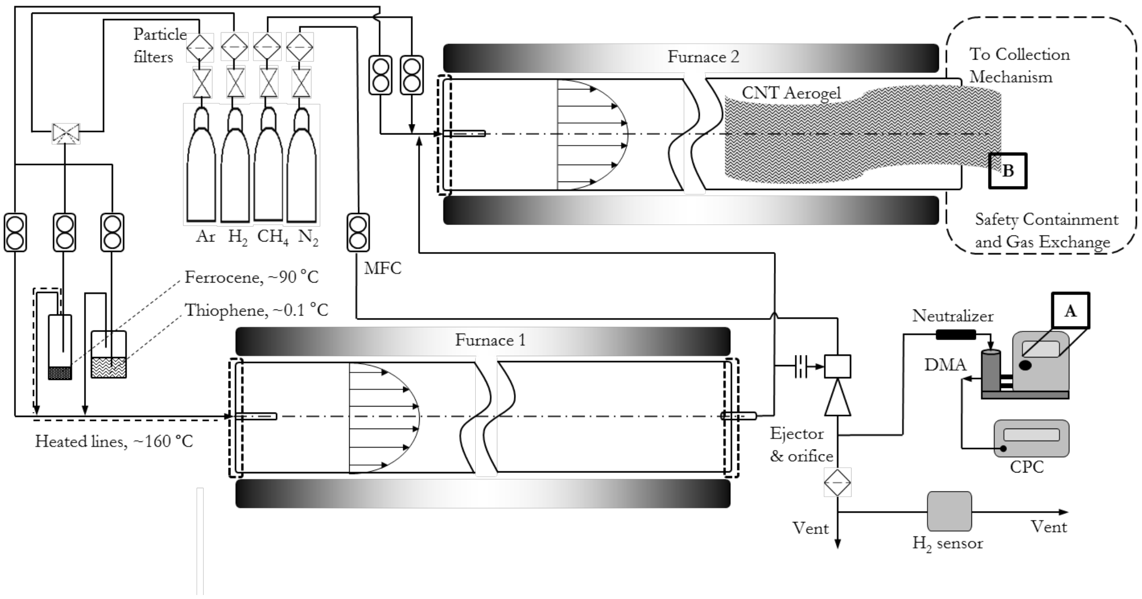

Figure S7. Detailed Schematic of Experimental Set-Up

This detailed schematic shows all the components used in our experimental work. Ferrocene and thiophene are supplied, in H_2, to Furnace 1 where catalyst nanoparticles are synthesized. Varying the temperature of Furnace 1 between 850 – 1150 °C allowed control of the catalyst particle size distribution. At the outlet from Furnace 1, samples from the catalyst nanoparticle stream can be diluted with N_2 allowing characterization of the particles using an SMPS system including a Condensation Particle Counter (CPC) and Differential Mobility Analyzers (DMA). The H_2 sensor is used to check that the sample dilution ratio is sufficient to reduce flammability of the sample stream below the lower explosion limit of H_2 (4%).

The catalyst nanoparticle stream from Furnace 1 is fed into Furnace 2, set at 1290 °C, with the co-addition of CH_4 and more H_2 if desired. CNT aerogels are synthesized and continuously collected on a winder from Furnace 2 through a gas exchange valve.

S8 – Classical Nucleation Theory - Explanation of a Critical Radius[20]

Before any coagulation can occur, in for example the FCCVD reactor, gas-phase synthesis of nanoparticles must occur via nucleation. Particle formation can happen by homogeneous or heterogeneous nucleation with typical gas-phase synthesis processes being high-temperature processes, in which the nanoparticle melting point is exceeded and particles can be treated as droplets. Particle formation, as it occurs in the FCCVD reactor, starts with a vapour of evaporated precursors which evolves to a vapour of atoms via thermal decomposition of the precursors at elevated temperatures. Once the vapour reaches a point of sufficient supersaturation, homogeneous or, if any condensation nuclei are already present, heterogeneous nucleation occurs. The saturation ratio is strongly dependent on temperature; supersaturation is often achieved by rapid cooling (with a quenching gas, in our reactor system, the temperature gradient in the exit zone of the reactor serves the same purpose). During nucleation, the atoms (or molecules) undergo a phase transition. In the case of homogeneous nucleation, particles form from a supersaturated vapour without the assistance of nuclei whereas in case of heterogeneous nucleation, particle formation is promoted by condensation nuclei. In homogeneous nucleation, it is important to determine a so-called *critical diameter* for particle formation which is determined by the underlying thermodynamics. Furthermore, the actual nucleation rate will depend on the thermodynamics, supersaturation, reaction and cooling forces.

It is important to determine an expression that relates the concentration of a species (*e.g.* iron atoms) with a particle or droplet (condensed material) of radius d_p at an equilibrium. For a flat surface and interface, these values (vapour pressures) at given temperatures and pressures are well tabulated. However, for a curved interface and the consequent critical nuclei radius for particle nucleation the so called Kelvin effect needs to be taken into account. Considering the change in Gibbs free energy during the formation of a single droplet (or embryo) of pure material (*e.g.* iron) of diameter d_p containing g atoms (or molecules) of the material gives

Equation 1

$$\Delta G = G_{embryo} - G_{pure\ vapour}$$

[20] Seinfeld, J. H. Atmospheric Chemistry and Physics of Air Pollution. 1986, 738.

Let n_T be the number of vapour atoms (or molecules). Once the embryo forms, the number of vapour atoms is reduced to $n=n_T-g$; including the surface energies, this leads to

Equation 2

$$\Delta G = n \cdot G_v + g \cdot G_l + \pi \cdot d_p^2 \sigma - n_T \cdot G_v = g(G_l - G_v) + \pi \cdot d_p^2 \sigma$$

with G_v being the free energy of an atom (or molecule) in the vapour phase, G_l the free energy of an atom (or molecule) in the liquid phase and σ the surface energy. The embryo size can be reformulated using a volume v_l occupied by an atom (or molecule) in the liquid phase

Equation 3

$$\frac{\pi \cdot d_p^3}{6} = g \cdot v_l$$

and therefore

Equation 4

$$\Delta G = \frac{\pi \cdot d_p^3}{6 v_l}(G_l - G_v) + \pi \cdot d_p^2 \sigma$$

$G_l - G_v$ is a change in Gibbs free energy and can be described by

Equation 5

$$dG = Vdp - SdT$$

and for isothermal conditions as

Equation 6

$$dG = Vdp = (v_l - v_v)dp$$

Since $v_l << v_v$ Equation 6 becomes

Equation 7

$$dG = Vdp \approx -v_v dp$$

Using the ideal gas law v_v can be written as

Equation 8

$$v_v = \frac{V}{nN_A} = \frac{k_B T}{p}$$

which leads to

Equation 9

$$dG = \approx -v_v dp = -k_B T\frac{dp}{p} = -k_B T\int_{p_{m0}}^{p_m}\frac{dp}{p} = -\,k_B T ln\frac{p_m}{p_{m0}} = -k_B T ln S_r$$

where p_m is the vapour pressure of the material, p_{m0} the equilibrium vapour pressure of the material over a surface (note for a flat surface $p_m = p_{m0}$) and S_r is the saturation ratio. This then gives the final equation for the change in Gibbs free energy during embryo formation:

Equation 10

$$\Delta G = -\frac{\pi \cdot d_p^3}{6v_l}k_B T ln S_r + \pi \cdot d_p^2\sigma$$

The first part of this equation is the volume free energy of the embryo and the second part is the surface free energy. The implications of Equation 10 are illustrated in Figure S8. It shows the reconciliation between the energy increasing, as a consequence of the increase in surface energy, and the energy decrease, due to the formation of a lower energy phase. If S_r>1, the surface energy dominates. For larger droplet diameters, the volume free energy of the embryo becomes increasingly important. Once the droplet reaches a critical size (d_p^*) the droplet can grow in size; any droplet smaller than this critical droplet size will evaporate and shrink. The critical droplet size is given as

Equation 11

$$d_p^*\Big|_{\frac{\partial\Delta G}{\partial d_p}=0} = \frac{4\sigma v_l}{k_B T \cdot ln S_r}$$

and relates the critical diameter of a droplet to the physical properties of the substance and the saturation ratio of its environment. Equation 11 is additionally referred to as the Kelvin equation.

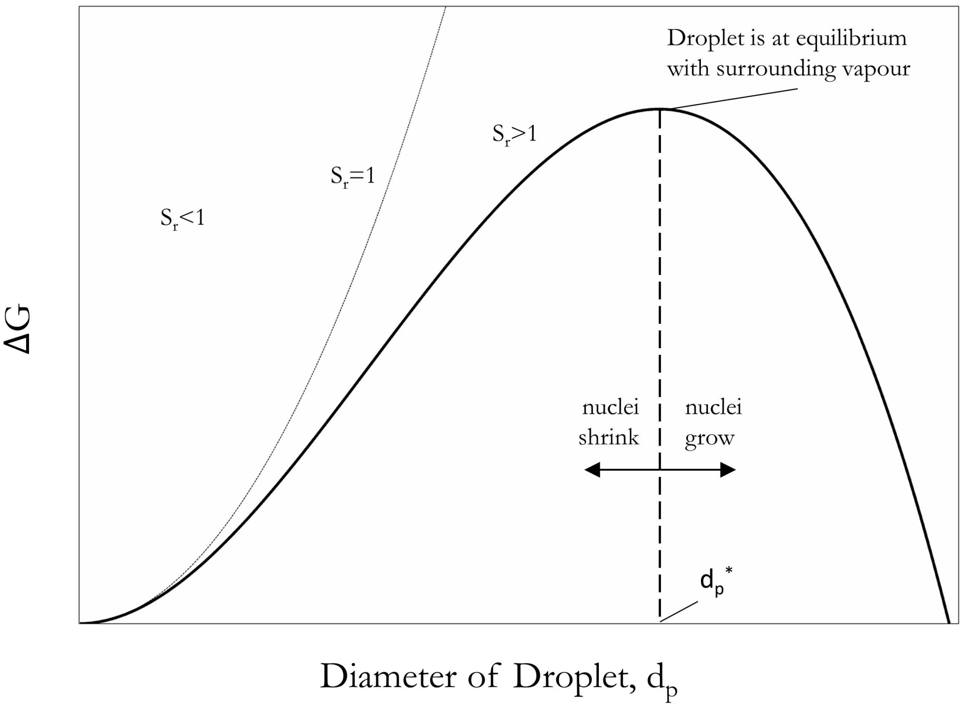

Figure S8. Slope of the Gibbs free energy during droplet formation as a function of droplet size for a saturation ratio > 1. d_p^* is the critical droplet size. Smaller droplets will evaporate and only droplets of this critical size will continue to grow.

S9 - Estimation of Particle Losses within Reactor

Particle penetration, indicating the proportion (0 – 100%) of the different sized nanoparticles that get through the reactor without being lost to the walls, is shown in Figure S9. Coagulation and any other particle dynamics apart from diffusion and thermophoresis are omitted for particles entering the reactor tube. Furthermore, a uniform temperature profile along the reactor axis is assumed.

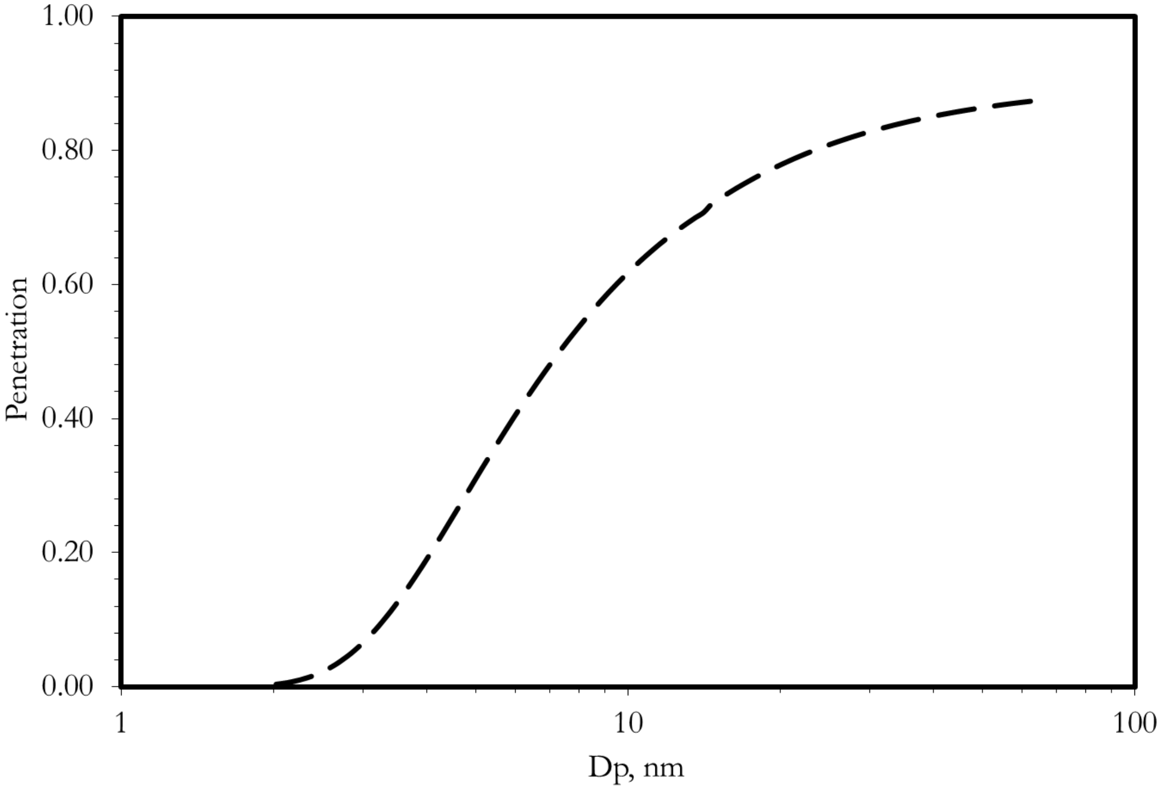

Figure S9. Estimation of the particle diffusion and thermophoretic losses within the reactor. The dependence of penetration through the reactor tube on the nanoparticle diameter using 0.8 slpm bulk H_2-flow with an average temperature of 1000 °C is shown. Thermophoresis is taken into account as an average 150 °C difference between the reactor wall and the actual gas flow. The penetration indicates the proportion (0 – 100%) of the different sized nanoparticles that get through the reactor without being lost to the walls. It is likely that Fe not homogenously nucleating with the help of sulfur will mainly be lost to the reactor walls (heterogeneous nucleation).

S10 – Raman analysis of CNT aerogels from different catalyst sources

Raman analysis of CNT material was conducted as described in the Methods section of the main paper. All show, predominantly, the typical features of MWNTs, with a D, G and G' peak. While the baseline in the 0-150 cm^{-1} region is not flat and may contain small contribution from some SWNT RBMs, the lack of any obvious G- contribution to the G peak and the relatively low G:D ratio indicates an overwhelming dominance of MWNTs.

S10a Standard continuously-spun aerogel, presented for comparison purposes

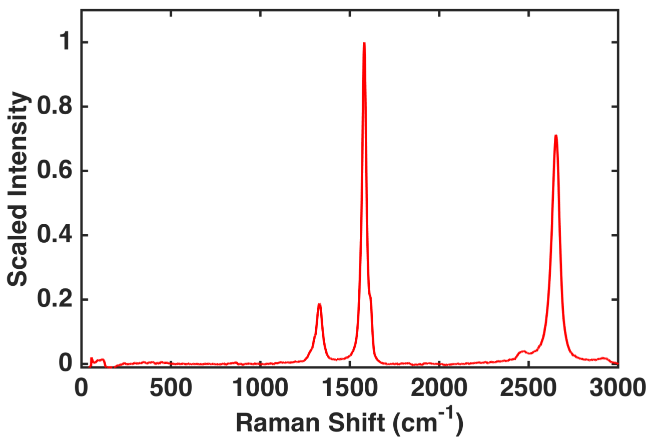

S10b Entangled CNT aerogel-networks synthesised using an iron plasma spark generator as a catalyst source

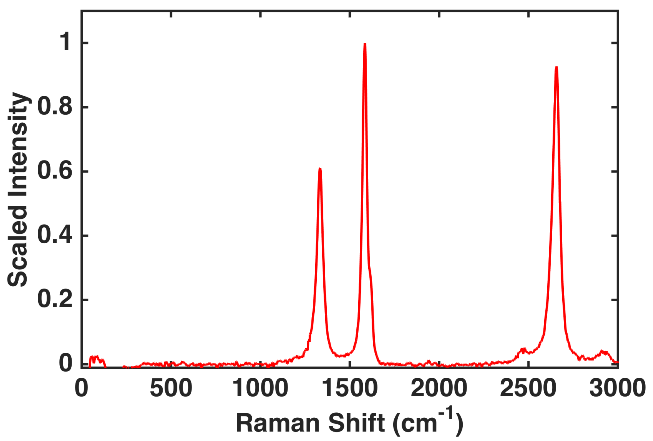

S10c Entangled CNT aerogel-networks synthesised using Cobaltocene as a catalyst source

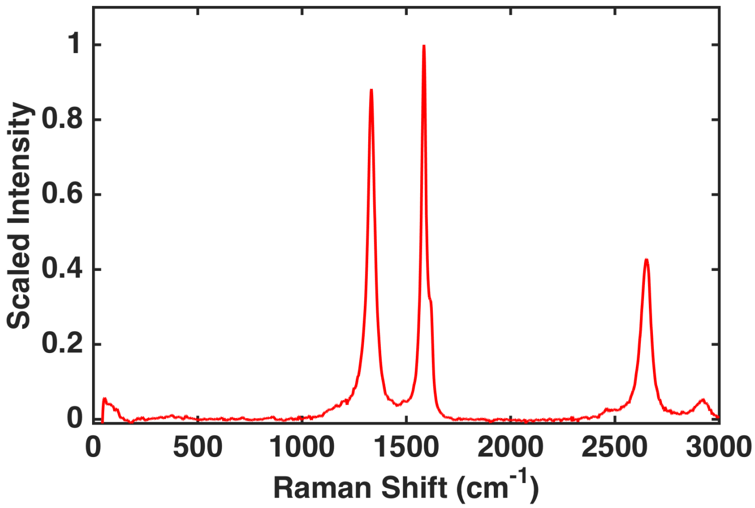

S10d Raman spectra of material containing small CNT clusters synthesised using Nickelocene as catalyst source.

The G:D < 1 and relatively small G' indicates the highly defective nature of the poorly formed CNT material present

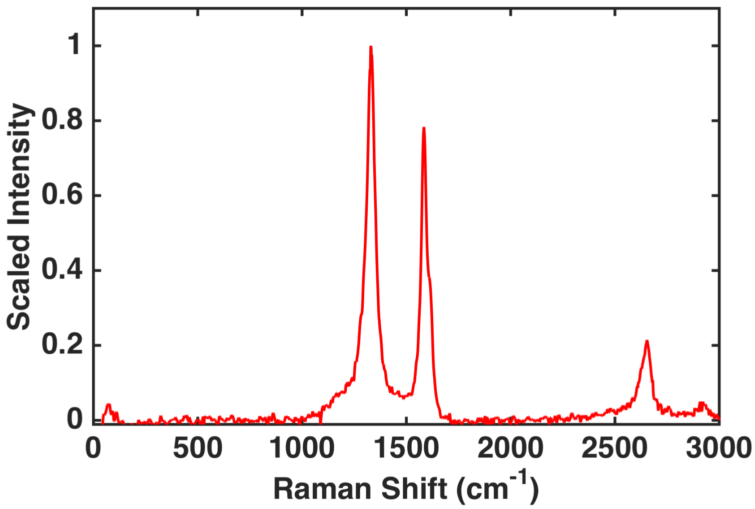

Supplement: Supplementary file 1 — Supplementary Information [file 41598_2017_14775_MOESM1_ESM.pdf]
